# Phytochemical Profile, Plant Precursors and Some Properties of Georgian Propolis

**DOI:** 10.3390/molecules27227714

**Published:** 2022-11-09

**Authors:** Piotr Okińczyc, Jarosław Widelski, Monika Ciochoń, Emil Paluch, Anna Bozhadze, Malkhaz Jokhadze, Gocha Mtvarelishvili, Izabela Korona-Głowniak, Barbara Krzyżanowska, Piotr Marek Kuś

**Affiliations:** 1 Department of Pharmacognosy and Herbal Medicines, Faculty of Pharmacy, Wrocław Medical University, Ul. Borowska 211a, 50-556 Wrocław, Poland; 2 Department of Pharmacognosy with Medicinal Plants Garden, Lublin Medical University, Ul. Chodźki 1 (Collegium Universum), 20-093 Lublin, Poland; 3 Department of Microbiology, Faculty of Medicine, Wroclaw Medical University, Tytusa Chałubińskiego 4, 50-376 Wrocław, Poland; 4 Department of Pharmacognosy, Tbilisi State Medical University, 33 Vazha-Pshavela Ave, 0186 Tbilisi, Georgia; 5 Department of Pharmaceutical Botany, Tbilisi State Medical University, 33 Vazha-Pshavela Ave, 0186 Tbilisi, Georgia; 6 First University Clinic, Tbilisi State Medical University, 33 Vazha-Pshavela Ave, 0186 Tbilisi, Georgia; 7 Department of Pharmaceutical Microbiology, Medical University of Lublin, 20-093 Lublin, Poland

**Keywords:** propolis, Georgia, UHPLC-DAD-MS/MS, antioxidant, antimicrobial, poplar, aspen, *Populus*, plant precursor

## Abstract

Propolis (bee glue) is a resinous substance produced by different species of bees i.a. from available plant resins, balsams, and exudates. It is characterized by significant biological activity (e.g., antimicrobial and antioxidant) and phytochemical diversity related to the available plant sources in specific geographical regions. The available scientific literature on propolis is quite extensive; however, there are only a few reports about propolis originating from Georgia. Therefore, our research was focused on the characterization of Georgian propolis in terms of phytochemical composition and antimicrobial/antioxidant activity. Performed research included UHPLC-DAD-MS/MS phytochemical profiling, determination of total phenolic and flavonoid content, antiradical and antioxidant activity (DPPH and FRAP assays) as well as antibacterial activity of propolis extracts obtained using 70% ethanol (70EE). Georgian propolis extracts exhibited strong activity against Gram-positive bacteria (22 mm—disc assay/64 µg/mL—MIC for *S. aureus*, sample from Imereti) and weaker against Gram-negative strains as well as strong antioxidant properties (up to 117.71 ± 1.04 mgGAE/g in DPPH assay, up to 16.83 ± 1.02 mmol Fe^2+^/g in FRAP assay for samples from Orgora and Qvakhreli, respectively). The phytochemical profile of Georgian propolis was characterized by the presence of flavonoids, free phenolic acids, and their esters. In most of the samples, flavonoids were the main chemical group (52 compounds), represented mainly by 3-*O*-pinobanksin acetate, pinocembrin, chrysin, galangin, and pinobanksin. The primary plant precursor of the Georgian bee glue is black poplar (*Populus nigra* L.) while the secondary is aspen poplar (*P. tremula* L.).

## 1. Introduction

Propolis is a bee product known for multiple healing properties [1,2,3]. It was proven, that propolis exhibits antimicrobial, antioxidative, anti-inflammatory as well as wound-healing activity [1]. One of the first reports on the medicinal properties of bee glue originated from Ancient Egypt and Greece. However, it is possible that propolis could have been in use much earlier, because the history of bee product usage may be tracked to c. 13,000 BC [4]. The first modern research on propolis started in the late 19th century and 20th century. Modern researchers confirmed the previous concept of ancient Roman philosopher Plinius the Elder, that propolis originated from plant resins and exudates, especially from buds [1]. Moreover, it was also revealed, that bees prefer to collect resins from specific plant species. Therefore, usually exudates of one plant species dominate over the other. It is possible to divide propolis into several types and subtypes due to the presence of resins of different plant precursors. In the temperate hemisphere, *Apis mellifera* L. collects mainly exudates and resins from poplars (*Populus* genus plant precursor) and birches (*Betula* genus plant precursor). Usually, black poplar—*P. nigra* L. or poplars with similar resin composition, e.g., *P. balsamifera* and *P. gileadensis,* are species preferred by bees [4]. Such propolis is described as black poplar type (*P. nigra* composition) and the other common type characterized by different chemical composition is aspen propolis (deriving from *P. tremula* L.) [5,6]. Another type is birch propolis that in pure form is more often present in colder areas, where poplar trees are not present [5,6]. In warmer climates where poplar trees are also absent, bees are forced to use other plant sources to form bee glue. One of the most known tropical plant precursors of propolis is *Baccharis* and *Dalbergia* genera [7]. The chemical composition of propolis originating from different flora varies, but its general profile of biological activity remains very similar. This phenomenon is probably connected with the propolis function in the bee hive, that most probably determines the specific bees’ preferences for botanical sources [2,6,8]. However, notable differences are usually [5,8] observed in the level of activities [7,9]. These differences are connected with the plant origin of propolis, which is crucial for its medicinal value [6,10]. Additionally, the geographical origin of propolis may also have an impact on chemical composition and activity due to the occurrence of different chemotypes of the plants in the specific area [5,8]. *Populus* trees are known for their extensive interspecific hybridization [11,12] as well as for variability in chemotypes of exudates [5,8]. Moreover, sometimes observed discrimination of poplar resins by honey bees [13] is an additional reason for detailed phytochemical screening of propolis from different geographical regions despite wide research of poplar propolis around the world. In Georgia country, the *Populus* genus is widely spread (especially *P. nigra* [14] and *P. tremula* [15]) as well as *Betula* (mainly *B. pubescens* [16] and *B. pendula* [17]). For this reason, it is expected that Georgian propolis may originate from black poplar. Moreover, one minor report [18] described the presence of *P. nigra* chemical markers in propolis from Armenia and Georgia. Propolis from the Caucasian country Georgia is not yet been fully investigated [18,19,20], especially in terms of phytochemical composition. Previous works include GC/MS analysis of silylated extracts, only of single propolis samples from Georgia and Armenia [18]. Gabunia et al. determined also antimicrobial activity of Georgian propolis against *Candida*, *Escherichia coli*, *Staphylococcus aureus*, *Streptococcus pyogenes*, and *Streptococcus fecalis* (diffusion method) and the relation between optical density and antimicrobial activity [19]. Aladshvili et al. reported a study on the correlation between polyphenol content and antioxidant activity (determined by spectrophotometry) of Georgian propolis [20]. Preliminary research on this topic was performed also as a part of a master thesis performed in our group [21].

Literature data exhibited, that propolis of poplar, aspen, and birch origin is expected as a strong antimicrobial [5,10] and antioxidant agent [22,23]. A typical standard in propolis research is the investigation of 70% aqueous ethanol propolis extracts (70EE) [3]. The popularity of 70EE research is caused by the common usage of this type of extract in traditional medicine and its proven efficiency in optimal extraction of flavonoids as well as providing the highest antimicrobial activity [24]. For these reasons, the scope of the current study was to focus on: (i) detailed UHPLC-DAD-MS/MS profiling of 15 different 70EE of Georgian origin from different locations as well as (ii) evaluation of their antimicrobial potential using both Kirby–Bauer disc diffusion method as well as determination of MIC, (iii) and determination of classical antioxidant activity (DPPH and FRAP tests which base on donor electron mechanism). Obtained data were further used to determine potential plant precursors of Georgian propolis and the connection between biological activity and plant origin.

## 2. Results and Discussion

### 2.1. Propolis UHPLC-DAD-MS/MS Profile and Component Identification Procedures

**Figure 1:**
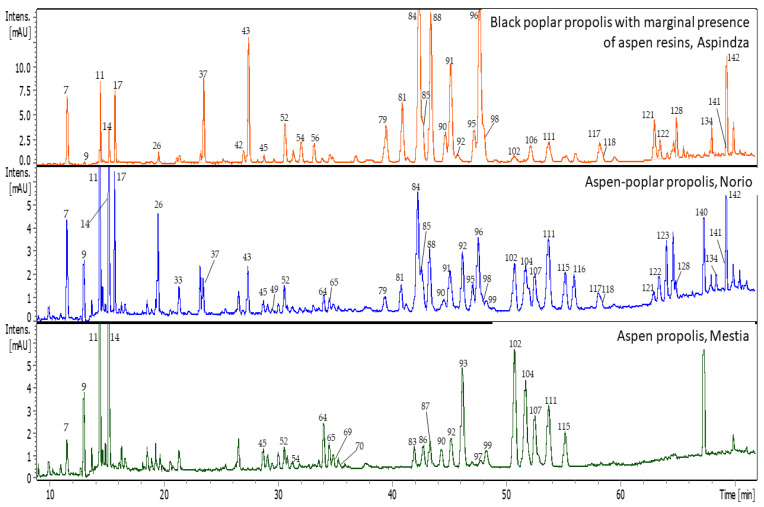
Representative UHPLC-DAD chromatograms of Georgian types of propolis at 280 nm.

Propolis is a complex natural matrix containing numerous components and the biggest challenge is posed by the samples with mixed plant origin containing many components with similar affinity to stationary phases such as phenolic acids glycerides, monoesters, and some flavonoid aglycones. During previous research we used different reverse-phase type columns such BEH C18, (1.7 µm, 130 Å, 2.1 × 150 mm) (Waters, Milford, CT, USA) [10,22], Kinetex^®^ F5 (2.6 μm, 100 Å, 150 × 2.1 mm) [23,25] and Kinetex^®^ C18 (2.6 μm, 100 Å 150 × 2.1 mm) [21]. In the current research, we performed UHPLC-DAD-MS/MS analyses using Kinetex^®^ core-shell Polar C18, reverse-phase type column (2.6 μm, 100 Å, 150 × 2.1 mm). Among all the used columns in different investigations, the best separation was obtained in the current research. The UHPLC-DAD-MS/MS analysis based on separation on Kinetex^®^ core-shell Polar C18 allowed to detect 151 compounds in the ethanolic extracts of 15 propolis samples of Georgian origin (Table 1 and Table 2). Representative UHPLC-DAD chromatograms of different types of propolis are presented in Figure 1.

Presented MS/MS fragmentation spectra are obtained as results of deconvolution using the DataAnalysis software algorithm. Most of the components were identified by comparison with data reported in previous papers [8,10,22,25]. Basic parameters used for identification included UV and MS/MS spectra of chromatographic peaks as well as exact mass. For LC-MS/MS analysis of polyphenols, it is usually sufficient to use only electrospray negative ionization mode due to good ionization of polyphenols as well as to avoid ionization of artifact components. However, in propolis, there are present polyphenols that did not produce ions in negative mode or produce just trace amounts. For example, these components are some flavonoids (tectochrysin and pinostrobin) [22,23] and phenolic acid esters (ferulic acid benzyl ester) [22,23]. They are relevant components of propolis [5,22,23] and poplars resins [5,35,36]. Therefore, MS detection in both ESI-NEG (electrospray-negative mode) and ESI-POS (electrospray-positive mode) is important.

**Figure 2:**
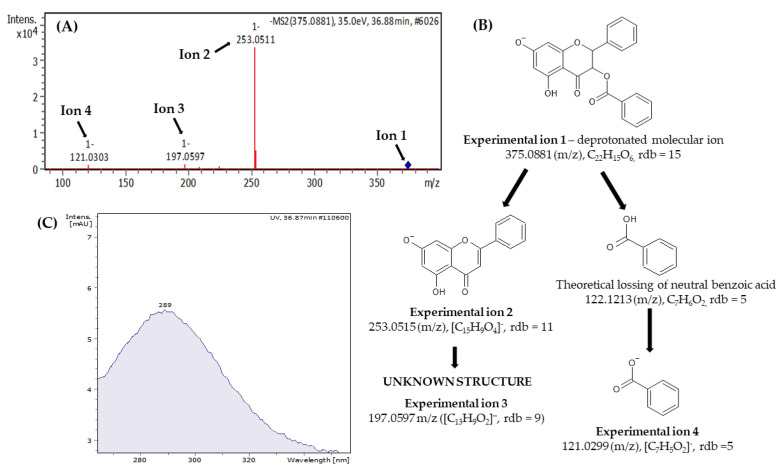
Experimental MS/MS (**A**) and UV (**B**) spectrum of 3-*O*-pinobanksin benzoate and proposed fragmentation scheme (**C**).

The samples of Georgian propolis contained typical compound groups for this type of product such as flavonoids, free phenolic acids, and their monoesters as well as glycerides. Among most of the analyzed propolises, flavonoids were the main chemical group (52 substances). In this group, the largest peaks were observed for 3-*O*-pinobanksin acetate, pinocembrin, chrysin, galangin, and pinobanksin. Most of the identified flavonoids were aglycones, and only one glucoside was observed (apigetrin, 7-*O*-apigenin glucoside). The procedure of the component identification presented an example of pinobanksin-3-*O*-benzoate, the rarer ester of pinobanksin. Its spectra as well as possible fragmentation patterns are presented in Figure 2. Previously, it was observed in *P. deltoides* exudates [37] and propolis samples from the United Kingdom [31]. This compound was characterized by a UV spectrum shape corresponding to other pinobanksin esters and maximum absorbance at 289 nm [22,38]. Pinobanksin-3-*O*-benzoate has a monoisotopic molecular mass of 376.3588 [C_22_H_16_O_6_]. During the experiment, a deprotonated molecular ion with about 375.0881 *m*/*z* value was observed. Mass calculation exhibited its deprotonated molecular ion [M-H]^−^ formula as [C_22_H_15_O_6_]^−^ and 15 rdb (ring and double bond equivalents). This ion produced daughter fragments 253.0515 *m*/*z* ([C_15_H_9_O_4_]^–^, rdb = 11), 197.0597 *m*/*z* ([C_13_H_9_O_2_]^–^, rdb = 9) and 121.0299 *m*/*z* ([C_7_H_5_O_2_]^–^, rdb = 5). Loss of fragment [C_7_H_6_O_2_] (calculated mass 122.0368) corresponds to benzoic acid and results in fragment 253.0515 *m*/*z* ([C_15_H_9_O_4_]^–^. Probably, the first one re-ionized to benzoic acid anion that corresponded to fragment [C_7_H_5_O_2_]^–^ observed during the experiment. Fragment 253.0515 *m*/*z* [22,38] is characteristic of pinobanksin-3-*O*-esters as well as pinobanksin. It is usually interpreted as dehydrated and deprotonated pinobanksin molecular ion [C_22_H_16_O_6_–H–H_2_O]^–^. Since position 3 in pinobanksin does not have a double bond, it is the most sensitive to dehydration. For this reason, the presence of ion 253.0515 *m*/*z* determined the esterification position in pinobanksin as 3. Apart from fragment 253.0515 *m*/*z*, ion 197.0597 *m*/*z* is also observed for pinobanksin [39] as well as their esters, but not always [22,38]. In our results, it was always observed in the MS/MS spectra of pinobanksin and their known esters. Therefore, in our opinion, its notable presence probably depends also on the used ionization parameters. Generally, pinobanksin-3-*O*-esters produced also other fragments (e.g., deprotonated pinobanksin molecular ion and next pinobanksin fragments), but heavier esters produce lower amounts of daughter ions [38,40].

The second considerable group in the number of components was phenolic acids monoesters (23 components) with caffeic acids monoesters as dominant components. Among most of the samples, prenyl and isoprenyl caffeic acids esters as well as cinnamyl ester of cinnamic acid were the main components in this group. Only in samples PAS, MES, and KAK relevant presence of caffeic acid phenethyl ester (CAPE) was observed.

**Figure 3:**
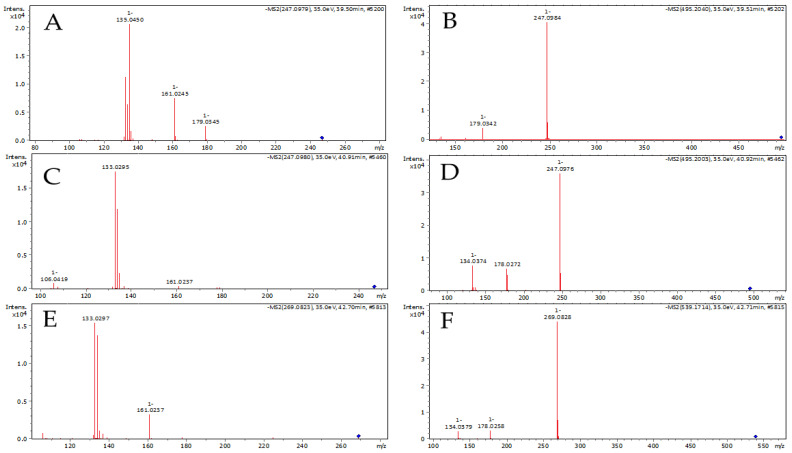
Experimental MS/MS spectra of caffeic acid esters and corresponding dimeric forms. Caffeic acid 2-methyl-2-butenyl ester (**A**) and its dimer (**B**); caffeic acid 3-methyl-2-butenyl ester (**C**) and its dimer (**D**); caffeic acid benzyl ester (**E**) and its dimer (**F**).

Most of the components in this group exhibited relatively similar patterns of mass fragmentation—loss of alcohol from the esters structure and further fragmentation of free phenolic acid structure [38,41]. They are usually described in the literature as caffeic acid prenyl or isoprenyl esters without further identification [38,42]. However, there are two different components described such as caffeic acid isoprenyl ester—caffeic acid methylbutenyl ester [43] and caffeic acid methylbut-di-enyl ester [44]. The first component contains one double bond in the aliphatic chain, while the second has two. Both structures should produce different deprotonated molecular ions in electrospray negative mode—247 *m*/*z* [C_14_H_15_O_4_]—for caffeic acid methylbutenyl ester and 245 *m*/*z*, [C_14_H_13_O_4_]—for caffeic acid methylbut-di-enyl ester in negative ionization mode. Therefore, numerous research [8,38,40,41,45] described only deprotonated molecular ion 247 *m*/*z* (or corresponding molecular mass 248) these components were different isomers of caffeic acid methylbutenyl but not methylbut-di-enyl. This inconsistency was contained in numerous research by repetition. In our opinion, it is better to use the name “prenyl caffeates/caffeic acid esters” for different methylbutenyl isomers, than isoprenyl, because this allows avoiding inconsistency. However, there are some papers which describe the full identification of methylbutenyl caffeates in propolis [5,46,47] or poplar resins [5]. Cited papers described three main esters—3-methyl-2-butenyl caffeate, 2-methyl-2-butenyl caffeate, and 3-methyl-3-butenyl caffeate [5,46,47]. Experimental fragmentation of methylbutenyl and benzyl esters as well as dimeric form are presented in Figure 3, and they will be further discussed. In the investigated samples, three peaks characterized by deprotonated molecular ions about 247.0985–247.0989 *m*/*z* [C_14_H_15_O_4_]^–^ and a UV maximum of 325 nm were present (Table 1). These parameters corresponded to isomers of caffeic acid methylbutenyl (prenyl) esters [10,22,46]. Gardana and Simonetti [46] described the main product of 3-methyl-3-butenyl caffeate fragmentation as 179 *m*/*z* and 135 *m*/*z* (experimental MS/MS spectra in Figure 3A), while 3-methyl-2-butenyl and 2-methyl-2-butenyl caffeates should rather produce 178 *m*/*z* and 134 *m*/*z*. This difference is a result of the presence allyl group close to the ester group in 3-methyl-2-butenyl and 2-methyl-2-butenyl caffeates. In our experiment, ion 133 *m*/*z* was the main fragment in previous MS/MS fragmentation spectra of 247 *m*/*z* (Figure 3C) instead of expected fragments 178 *m*/*z* and 134 *m*/*z*. However, when the deconvolution algorithm was used for MS/MS fragmentation spectra, the fragment of 134 was selected as the main as well as the fragment of 247 *m*/*z*. According to the literature data [46], fragment 133 *m*/*z* is a product of further fragmentation of 134 *m*/*z* or 135 *m*/*z*. Under this concept, 178 *m*/*z* and 134 *m*/*z* are anion radicals, while fragment 133 *m*/*z* is an anion. Therefore, it was probable that unstable 178 *m*/*z* and 134 *m*/*z* quickly produced more stable 133 *m*/*z* in the used MS/MS condition. In the experiment of Gardana and Simonetti [46], the fragmentation pattern of 3-methyl-2-butenyl and 2-methyl-2-butenyl caffeates was visible only in low collision energy parameters, while other researchers did not describe differences in the ionization of methylbutenyl caffeic acid esters [22,40]. Moreover, the production of the main ion 133 *m*/*z* was also observed for caffeic acid benzyl ester (Figure 3F) which also should produce mainly radical anions [46]. In our opinion, the critical point of identification is the usage of proper negative electrospray parameters. Our hypothesis is supported by the fact that in the MS conditions used we observed also the production of dimers of methylbutenyl esters of caffeic acid—495.2043 *m*/*z* ([C_28_H_32_O_8_]^–^, Figure 3B,D). Dimeric form of suspected 3-methyl-2-butenyl (Figure 3D) produced ion 247 *m*/*z*, 178 *m*/*z* and 134 *m*/*z* while 3-methyl-3-butenyl caffeate was reduced to 247 *m*/*z* and 179 *m*/*z* fragments (Figure 3B). Anion radical ions were also noted for MS/MS spectra of caffeic acid benzyl ester (Figure 3F). This difference corresponded to the patterns of Gardana and Simonetti [46]. The observed fragmentation pattern allowed distinguishing 2-methyl-2-butenyl caffeate and 3-methyl-2-butenyl caffeate from 3-methyl-3-butenyl caffeate. Further identification of 2-methyl-2-butenyl and 3-methyl-2-butenyl caffeates was performed due to differences in their concentration in propolis [5]. Among these esters, the largest concentration is usually described for 3-methyl-2-butenyl while 2-methyl-2-butenyl caffeate exhibit about times lower concentration [5,24]. For this reason, the higher UV peak with corresponding ions 247 *m*/*z* and related 133 *m*/*z* (or 134 *m*/*z*) should be 3-methyl-2-butenyl ester, while lower 2-methyl-2-butenyl. Moreover, 2-methyl-2-butenyl ester of caffeic acid did not produce a dimeric form due to too low concentration.

Besides caffeic acid derivatives, methylbutenyl (or prenyl/isoprenyl) esters of *p*-coumaric acid are also present in propolis [45]. Similarly to caffeic acid 3-methyl-2-butenyl, 2-methyl-2-butenyl and 3-methyl-3-butenyl are reported as main esters. In our opinion, a similar identification procedure as for caffeates may be used for *p*-coumaric acid esters. As a result, 3-methyl-2-butenyl and 2-methyl-2-butenyl *p*-coumarates should exhibit radical fragmentation in MS/MS, while 3-methyl-3-butenyl rather produces no radical ions. However, the difference in concentration of 3-methyl-2-butenyl and 2-methyl-2-butenyl *p*-coumarates is considerable in poplar resins [34], but not in propolis [45]. Therefore, distinguishing between these two components is not so reliable such as caffeic acid esters.

The third group of compounds—phenolic acids glycerides—consisted of 18 components. In comparison with flavonoid and hydroxycinnamic monoesters, glycerides were less represented, also in terms of peak sizes. Among observed glycerides, the largest peaks belonged to 2-acetyl-1,3-di-*p*-coumaroylglycerol; however, the most often caffeoylglycerol was observed. In the case of phenolic acids glycerides, the situation is complicated due to different glycerol substitution positions. For example, acetyl-di-*p*-coumaroylglycerol is presented in two position isomers, 2-acetyl-di-1,3-*p*-coumaroylglycerol and 1-acetyl-di-2,3-*p*-coumaroylglycerol. Moreover, 1-acetyl-di-2,3-*p*-coumaroylglycerol has asymmetric carbon in the glycerol chain and may be presented in two tautomeric forms. Different optical isomers were also possible for many other glycerides. However, previous research exhibited that symmetric forms of glycerides dominate over non-symmetric (e.g., 1,3-di-caffeoylglycerol had a stronger concentration than 2,3-di-caffeoylglycerol) [28,46]. For this reason, it is possible to identify the position isomers of phenolic acid glycerides. Similar research is not known for different tautomeric forms, therefore distinguishing between them is not possible at this moment.

In the case of free phenolic acids, all the samples exhibited the presence of caffeic, cinnamic, ferulic, and isoferulic acids. Among them, ferulic and isoferulic acids usually are present less frequently than caffeic and *p*-coumaric acids.

The samples contained also some other components such as cinnamic acid, vanillin, caffeoylquinic acid, and some unidentified components. Most of them were represented by small peaks in the chromatograms.

### 2.2. Plant Origin of Georgian Propolis

Performed UHPLC-DAD-MS/MS analyses exhibited the presence of flavonoids, phenolic acids monoesters, and glycerides as well as free phenolic acids as main components. Among these components, large peaks that can be related to propolis plant precursor markers were observed, which is further discussed.

Black poplar (*P. nigra*), aspen poplar (*P. tremula*), and Birch genus had their own specific markers and their presence is the most important to confirm propolis plant precursor. However, it is necessary to add, that some components are common for black poplars, aspens, and birches. These common components mainly included some flavonoid aglycones and other components. For example, sakuranetin is presented in poplars, aspens, and birches exudates while kaempferide and acacetin are rather characteristic for aspen and birch [22]. A similar situation is also with *p*-coumaric acid benzyl ester, which is observed in both *P. nigra* and *P. tremula* [8]. Besides the presence of common components, important is also their concentration—black poplars are known for their relatively high presence of free phenolic acid, while in aspen they are minor components and present as traces or absent in birches [5].

Most of the analyzed Georgian propolises (10 samples) exhibited characteristic UHPLC-DAD-MS/MS profile for black poplar origin, pronounced presence of *P. nigra* markers peaks, and lack of other specific markers. Observed black poplar markers included flavonoids (chrysin, pinocembrin, galangin, and pinobanskin with its esters, especially 3-*O*-pinobanksin acetate) [5,22,36] as well as phenolic acids monoesters (mainly ester of caffeic acids such as 3-methyl-2-butenyl, 2-methyl-2-butenyl, and phenethyl) [5,22].

In four samples (ASP, MTS, NOR, U.R.1, and U.R.2) besides large peaks of compounds related to *P. nigra*, smaller peaks of compounds that may be related to aspen origin were observed. In the PAS sample, the peaks related to aspen and black poplar were present at similar levels, while in MES propolis *P. tremula* peaks dominate over *P. nigra*. Observed specific components for aspen origin were phenolic acids glycerides, especially 2-acetyl-1,3-di-*p*-coumaroylglycerol (lasiocarpin A) [5,22]. Besides *P. tremula*, phenolic acids glycerides are also present in Asian poplars such as *P. lasiocarpa* [48] and *P. szechuanica* [5]. Interestingly, caffeoylglycerol was presented in almost all samples but often it was the only glyceride derivative in the sample; therefore, its presence was probably not connected with aspen origin. Apart from phenolic acid glycerides, typical for aspen resin is also the dominance of ferulic acid over isoferulic acid [5] while poplars usually contain more isoferulic acid than ferulic acid.

In temperate climate zones, propolis that is not derived from black poplar (or only partially derived from black poplar) is usually present in mountains or other areas with unfavorable microclimates for poplars. However, sometimes, local honey bees may discriminate against foreign black poplar chemotypes and prefer collecting exudates from other plant precursors [13]. For this reason, the same presence of *P. nigra* is insufficient to confirm the black poplar origin of propolis, and phytochemical analysis is required. In the current research, besides the phytochemical profile, distribution maps of *P. nigra* [14] and *P. tremula* [15] were used to determine potential propolis plant precursors.

Apart from the Populus genus, Georgian propolis contained also components whose presence may be connected with non-poplar origin. These substances included Betula genus markers (ermanin, acacetin, sakuranetin [22]) as well as unknown (e.g., caffeoylquinic acid in MTS). However, some of the known Betula markers are also Populus markers (sakuranetin, acacetin) [5,22] and only their dominance over Populus markers may determine Betula origin. Additionally, the sample from MES contained also quite large peaks of unidentified ermanin isomer (component 93 in Table 2 and Figure 1) which potentially may be pectolinaringenin (additional birch marker [5]). On the other hand, the marked presence of free phenolic acids (caffeic, *p*-coumaric, ferulic, and isoferulic) rather proves dominant Populus origin, because they are absent or only trace components in birches resins (about 2.5%) [5]. Therefore, all described components exhibited low concentrations in Georgian propolis, non-Populus resins were rather marginal plant precursors for Georgian propolis.

### 2.3. Total Phenolic and Flavonoid Content in Georgian Propolis and Classical Antioxidant Activity

In the current research, colorimetric assays were performed on re-dissolved dried propolis extracts and then calculated for crude propolis. For this purpose, we used extraction efficiency value. Extraction efficiency varied from 24.61% (PAS) to 57.93% (ORG). Most of the propolis extracts exhibited quite a high efficiency (between 30% and 40%) and four large efficiencies (almost 50% above 50%). Only three samples had extraction efficiency between 20 and 30%.

The results of colorimetric assays and extraction efficiency are presented in Table 3. Total phenolic content (TP) was from 27.39 ± 0.91 (NOR) to 126.77 ± 1.64 (VAR) mgGAE/g propolis (mg of gallic acid equivalents in g of crude propolis), while extracts contained from 89.88 ± 3.82 (ME) to 242.71 ± 3.12 (OTA) mgGAE/g extract (mg of gallic acid equivalents in g of dry extract). The highest amount of TP was observed in OTA, VAR, ORG crude propolis, and extracts from OTA, QVA, and VAR, respectively.

Flavonoid content (TF) varied from 7.57 ± 0.19 (ASP) to 67.16 ± 1.31 (MES) mgQE/g propolis (mg of quercetin equivalents in g of crude propolis). The same extracts exhibited amounts of flavonoids from 19.19 ± 0.48 (ME) to 125.50 ± 2.88 (VAR) mgQE/g extract (mg of quercetin equivalents in g of dry extract). TF was the largest in ASP, OTA, and VAR (crude propolis) as well as VAR, ASP, and AKH (extracts), respectively. The total phenolic and flavonoid content range was very similar to those observed for samples from various European and Asian countries [23,49,50].

All the samples contained low to quite high amounts of polyphenols in the calculation on crude propolis. However, the extracts exhibited moderately high to high amounts of polyphenols. Moreover, in most samples (8 from 14), flavonoids dominate over the rest of the polyphenols in colorimetric assays.

The antioxidant activity was determined in DPPH and FRAP assays. Generally, the antioxidant activity of natural components includes multiple effects which allow avoiding the overproduction and activity of reactive oxygen species (ROS) and further injuries of DNA and other macromolecules. In classical understanding, there are two main mechanisms of protection before oxidation—inactivation of ROS and avoiding ROS production [22,51]. DPPH is a test which describes the ability of ROS scavenging, while FRAP describes the ability to reduce Fe^3+^ to Fe^2+^ and avoid ROS production in the Fenton reaction [22,51]. Both tests are based on the antioxidant ability to electron donation on ROS (DPPH assay) or Fe^3+^ (FRAP). Today, it is questioned drawing too far-reaching conclusions about the antioxidant potential of natural substances based only on indirect methods such as DPPH [51]. Their limitation is mainly connected with different chemical structures from natural free radicals (DPPH) and too short a time of reaction (FRAP) [51]. However, they may be good predictors of antioxidant properties before using more expensive and complex tests due to their low cost as well as easy and fast procedures and the possibility of wide screening. For these reasons, DPPH and FRAP test was used in this paper.

DPPH values determined for Georgian propolis ranged from 13.48 ± 0.74 (PAS) to 68.19 ± 0.61 (ORG) mgGAE/g (mg of gallic acid equivalents in g of crude propolis). For extracts values from 47.880 ± 0.83 (IME) to 117.710 ± 1.04 (ORG) mgGAE/g (mg of gallic acid equivalents in g of dry extract). The strongest activity in the DPPH test exhibited ORG, QVA, and U.R.1 in the case of crude propolis while for extracts the most active samples were very similar—ORG, QVA, and U.R.2, respectively. Differences between U.R.1 and U.R.2 were low. The values were comparable to those observed in European propolis samples [23]

FRAP activity values ranged from 2.050 ± 0.00 (PAS) to 7.974 ± 0.002 (ORG) mmol Fe^2+^/g propolis (mmol of Fe^2+^ equivalents in g of crude propolis) while extracts activity varied from 7.77 ± 0.20 (KAK) to 16.83 ± 1.02 (QVA) mmol Fe^2+^/g extract (mmol of Fe^2+^ equivalents in g of dry extract). The largest values in FRAP assays were observed in ORG, OTA, VAR (crude propolis) as well as QVA, VAR, and OTA (extracts), respectively.

On the one hand, the colorimetric test exhibited that the lowest and the highest values were different for extracts and crude propolis but on the other hand, the highest values exhibited similar samples of crude propolis and extracts in this same test.

Statistical analysis exhibited different results for data calculated for crude propolis and extracts. In the case of crude propolis, every colorimetric assay was correlated with each other, while in the case of the extracts DPPH assays were not correlated with any other tests (Table 4). Moreover, most of the correlations of crude propolis were strong correlations (*p* < 0.01) and only the correlation of TF with DPPH exhibited a weaker correlation parameter (*p* = 0.027). The lack of correlation with DPPH for extracts is probably caused by too similar polyphenol concentrations and activity in the extracts. Extracts are concentrated isolated plant resins, while crude propolis contains plant resins “diluted” with wax and mechanical impurities. The correlation between extraction efficiency and DPPH confirmed our point of view. As a result, independent from amounts of plant resin content in crude poplar propolis, it should be expected strong radical scavenging activity of the balsam fraction itself. Therefore, a similar observation was not observed for FRAP tests, and it may be better to compare only the antioxidant activity of the extracts.

Interestingly, in our previous research on poplar propolis [22], we found a correlation between flavonoid and total phenolic content with DPPH tests while FRAP was not correlated with these values. In the literature, some researchers report a correlation between TP [52] and FC [53] with DPPH or not [54]. A similar situation was observed between FC and DPPH [54]. These differences were probably caused by different compositions of propolis as well as different protocols of DPPH tests used.

### 2.4. Antimicrobial Properties

Results of antibacterial assays, as well as statistical analyses, are presented in Table 5. In this study, we used the following reference bacterial (*Staphylococcus aureus*, *Enterococcus faecalis*, *Bacillus subtilis*, *Escherichia coli*, *Pseudomonas aeruginosa*, and *Klebsiella pneumoniae*) and fungal strains (*Candida albicans*, *C. glabrata*, *C. krusei*, and *Saccharomyces cerevisiae*) as well as drug-resistant Gram-positive bacteria—*S. aureus* MLS_b_ (macrolide-lincosamide-streptogramin B resistant *S. aureus*) and *S. aureus* MRSA (multi-drug resistant *S. aureus*). Our main purpose was the general screening of the antibacterial properties of Georgian propolis. For this reason, there were chosen strains with expected strong and weak resistance on propolis. Among Gram-positive, Georgian propolis was the most active against tested staphylococci species (Kirby–Bauer range from 13 to 22 mm and MIC from 512 to 64 µg/mL) and the weakest against *Enterococcus faecalis* (Kirby–Bauer range from 6 to 10 mm and MIC from 1024 to above 1024 µg/mL). It is interesting, that, some samples (MTS, KAK, AKH, DUS, IME) were more efficient against drug-resistant than non-resistant strains of staphylococci in the Kirby–Bauer method. However, in MIC tests, these differences were not always observed. In the case of fungi, the most sensitive species was *Saccharomyces cerevisiae* (Kirby–Bauer range from 10 to 16 mm and MIC from 1024 to above 1024 µg/mL) while *Candida krusei* exhibited the highest resistance (Kirby–Bauer range from 6 to 11 mm and MIC from 1024 to above 1024 µg/mL). The most resistant strains on propolis were Gram-negative bacteria. The activity was the weakest in terms of the Kirby–Bauer disc diffusion method (only 6 mm) and MIC (values above 1024 µg/mL).

Obtained antimicrobial activity profile is typical for 70EE of poplar propolis [10]. In terms of phytochemical composition, black poplar propolises contains three main chemical groups of components—flavonoids, free phenolic acids, and their monoesters) [10,55,56]. Research exhibited that proportion between these phytochemical groups is important for biological activity. The most researched is the connection between propolis components and its antimicrobial activity, especially antibacterial [10,55,56]. Some researchers exhibited a correlation between TP and propolis antibacterial activity [57], while others did not observe a link between these two properties [25]. Similar observations were also noted for TF. This parameter was correlated with antimicrobial activity [58] or not [59]. In our research, no correlation was observed between bacterial strains and TP and TF of extracts as well as crude propolises in Kirby–Bauer disc diffusion methods. However, correlations were observed in MIC assays but only for extracts. TF was correlated with *S. aureus* (reference), *S. aureus* MLS_b_, *S. aureus* MRSA P19, and *C. albicans*, while TP of extracts was not correlated with *S. aureus* MLS_b_, *S. aureus* MRSA P19, *C. albicans,* and *S. cerevisiase*. Therefore, all observed correlations for MIC were negative (R < 0), and both polyphenols and flavonoids exhibited a positive effect on the antibacterial activity of propolis. The lack of correlation in Kirby–Bauer assays may be explained by too low differences between samples. Interestingly, TF and TP exhibited a stronger correlation with drug-resistant *S. aureus* than non-resistant. Potentially, this may show, that creation of antibiotic resistance in *S. aureus* species accompanies lowering propolis polyphenol resistance. Moreover, the lack of correlation of TP and weak correlation of TF with non-resistant *S. aureus* may also support this hypothesis. However, this phenomenon requires further research.

Since propolis is more active against Gram-positive bacteria, *S. aureus* is a typical model organism for screening 70EE propolis antibacterial activity and searching correlations between its composition and antimicrobial activity. Research in this area proved that strong concentrations of free phenolic acid as well as single phenolic acid components exhibit a negative impact on antibacterial activity of propolis ethanolic extracts [10] or exhibit low impact [56]. An opposite effect was observed for some flavonoids as well as phenolic acid monoesters [10,56]. In the case of flavonoids, a positive impact on antimicrobial activity was exhibited usually by galangin [10,56], chrysin [10], pinocembrin [59], pinobanksin-3-*O*-acetate [10], and pinobanksin-5-methyl ether [10]. Among phenolic acid monoesters, the most important component is CAPE [56] as well as caffeic acid prenyl (3-methyl-2-butenyl) ester [56]. GC-MS research of Isidorov et al. exhibited, that *P. nigra* resins contain from 18.2 to even 42.5% of free phenolic acid and from 21.0 to 44.8% of flavonoid aglycones [5] as well as the different qualitative composition of resins [5,8]. The composition of phenolic acids monoesters was more stable (about 20%) [5]. For this reason, black poplar resins may be divided into a group with the dominance of free phenolic acids or flavonoid aglycones. Since an elevated amount of flavonoids is often positively correlated with the antibacterial activity of propolis, it is expected that propolis and poplars resins with the dominance of flavonoids are better antibacterial agents than ones with the dominance of free phenolic acids [24].

In previous research on the connection between antimicrobial activity and plant origin of propolis, there was proven that 70EE of poplar propolis usually exhibits higher activity than aspen ones [10]. Moreover, in the present research, the presence of aspen markers (phenolic acids glycerides) was connected with lower antibacterial activity. This observation was also noted in the present research, where samples with higher amounts of aspen resins and lower of poplar (NOR, ASP, MES) exhibited lower antibacterial potential against staphylococci species.

The strong activity of 70EE of Georgian propolis against staphylococci species determined their usage as a dermal medicament in traditional medicine [4,60]. The antimicrobial potential is one of the main components of wound treatment activity [60]. Moreover, it was proved that poplar propolis increases the healing of tissues as well as exhibits anti-inflammatory effects [60]. As a result, Georgian propolis of poplar origin may be potentially used as a burn and wound treatment agent.

## 3. Materials and Methods

### 3.1. Propolis and Reagents

Propolis samples from the following regions of Georgia were obtained in 2020: Aspindza, Norio, Pasanauri, Mestia, Orgora, Vardzia, Ota, Qvakhreli, and two unknown locations, while in 2021 samples were collected from Mtskhete, Kakheti, Akhatsikhe, Dusheti, and Imereti. Obtained propolis was frozen in liquid nitrogen and crushed in a mortar. Freezing and crushing procedures were repeated three times. Before extraction, ground propolis was stored in sealed containers at −20 °C.

LiChrosolv^®^ hypergrade eluents for LC-MS (acetonitrile, water, methanol), DPPH (2,2-diphenyl-1-picrylhydrazyl), TPTZ (complex of 2,4,6-tri(2-pyridyl)-s-triazine), iron(II) sulfate heptahydrate, and aluminium chloride hexahydrate were purchased from Merck company (Darmstadt, Germany). Folin–Ciocalteu reagent, ethanol (analytical grade) was purchased from ChemPur (Piekary Śląskie, Poland). Disodium hydrogen phosphate and sodium chloride were obtained from POCH (Gliwice, Poland). Mueller–Hinton agar and Sabouraud agar were obtained from Oxoid (Hampshire, UK).

### 3.2. Preparation of Extracts

Previously ground research material was extracted by ethanol in water (70:30; *v/v*) in proportion 1:10 (1.0 g of propolis per 10 mL of solution). Extraction was performed in an ultrasonic bath (Sonorex, Bandelin, Germany). Extraction conditions were set at 20 °C for 45 min and 756 W (90% of ultrasound bath power). Next, extracts were stored at room temperature for 12 h and then filtered through Whatman No. 10 paper (Cytiva, Marlborough, MA, USA). For all samples, extraction efficiency was calculated as the percent of dry extract mass in crude propolis.

### 3.3. UHPLC-DAD-MS/MS Profile of Propolis Extracts

Before analysis, 10 mg of propolis was dissolved in 10 mL and then filtered through a PVDF hydrophilic Alwsci^®^ 0.22 µm, Ø13 mm, membrane syringe filter (Alwsci, Hangzhou, China). Then, 1 μL of sample was injected into the Thermo Scientific™ UltiMate™ 3000 system (Thermo Fischer Scientific™ Dionex™, Waltham, MA, USA), equipped with an autosampler and DAD detector set at 280, 320, and 360 nm. Spectral data were recorded in the 200–600 nm range. Chromatographic separation was performed on Kinetex^®^ Polar C18 core-shell reverse phase column, 2.6 μm, 100 Å, 150 × 2.1 mm, column (Phenomenex, Torrance, CA, USA) with SecurityGuard^®^ ULTRA column with Polar C18, 4 × 2.0 mm cartridges (Phenomenex, Torrence, CA, USA) thermostated at 20 ± 2 °C. The mobile phase consisted of 0.1% formic acid in water (solvent A) and acetonitrile (solvent B). The flow rate was set at 0.4 mL min^−1^ and the separation was obtained using the following program of solvent B gradient: 5% at start and maintained isocratic to 5.0 min, increased to 10% in 5.1 min, and maintained isocratic to 10.0 min, increased to 20% in 10.1 min. and maintained isocratic to 13.1 min, increased to 30% in 30.7 min, 31% in 32.3 and maintained isocratic to 35.9 min and then increased to 32% in 38.0 min, 33% in 40.5 min, 34% in 47.0, 36% in 50.3 min, 40% in 55.5 min, 50% in 58.7 min, 75% in 71.0 min, 100% in 80.0 and maintained isocratic to 84.0 min and decreased to 5% in 88.0 min. At the end of the program, a 5% gradient of solvent B was maintained isocratic by 10 min to stabilize the column before the next injection.

UHPLC-DAD-MS/MS was performed using a Compact QqTOF MS detector (Bruker, Darmstadt, Germany). MS detector was used in electrospray negative mode. Parameters of analysis were: ion source temperature was set at 210 °C, nebulizer gas pressure was set at 2.0 bar, dry gas (nitrogen) flow 8.01 L/min, and temperature at 210 °C. The capillary voltage was set at 4.5 kV. The collision energy was set at 8.0 eV. Internal calibration was obtained using a 10 mM solution of sodium formate. For ESI-MS/MS experiments, collision energy was set at 35.0 eV and nitrogen was used as collision gas. The scan range was set from 30 to 1300 *m*/*z*.

### 3.4. Colorimetric Assays of Propolis Extracts

Colorimetric assays were performed using extracts described in the previous paragraph. Before proper measurements, preliminary analyses with different dilutions of basic extracts, from two to ten times, were carried out to obtain the most appropriate concentration for every assay.

Antiradical activity (DPPH Test), total antioxidant activity (FRAP Assay), total phenolic content (TP), and total flavonoid content (FC) assays were performed according to previously described methods [23] using dissolved dried extracts instead of previous propolis liquid extract. Every measurement was performed in triplicate. Results of DPPH and TP was presented as gallic acid equivalents per gram of crude propolis and its extracts, TF as quercetin equivalents per gram of crude propolis and its extracts, FRAP as mmol of Fe^2+^ quercetin equivalents per gram of crude propolis and its extracts. Every measurement was performed triple times. The standard deviation of measurements was under 5%.

### 3.5. Strains and Growth Conditions

In this study, we used the following bacterial (*Staphylococcus aureus* 25923, *S. aureus* MLS_b_, *S. aureus* MRSA P19, *Enterococcus faecalis* 29212, *Bacillus subtilis* 6633, *Escherichia coli* 25922, *Pseudomonas aeruginosa* 27853, and *Klebsciella pneumoniae* 700603) and fungal strains (*Candida albicans* 90028, *C. glabrata* 90030, *C. krusei* 6258, and *Saccharomyces cerevisiae* 3963). Bacterial strains were cultured in Mueller–Hinton II Broth BD (MHB) and fungal strains in MHB enriched with 2% glucose. The strains were incubated aerobically for 24 h at 37 °C (*E. coli*, *S. aureus* and *C. albicans*) or for 48 h at 28 °C for the remaining ones. Overnight microorganism cultures were centrifuged, washed with PBS (pH 7.4), and suspended in fresh MHB to obtain suitable optical density.

Microorganisms were obtained from the Department of Microbiology of Wrocław Medical University. All described strains were used in Kirby–Bauer disc diffusion and method minimal inhibitory and fungicidal concentrations assays.

### 3.6. Kirby-Bauer Disc Diffusion Method

Antimicrobial properties were determined with the disc-diffusion method according to the Clinical and Laboratory Standards [61]. All experiments were performed in triplicate.

### 3.7. Minimal Inhibitory and Fungicidal Concentrations

The values of the minimal inhibitory concentrations (MIC) were determined according to the modified protocol described before [29]. All experiments were performed in triplicate.

### 3.8. Statistical Analysis

Statistical analysis was performed in Statistica 14.0 software (StatSoft Power Solutions, Inc./Dell, Round Rock, TX, USA). Analysis based on matrix correlation with the evaluation of Pearson correlation and r parameters. The matrix was built of colorimetric test values (DPPH, FRAP, TP, and FC) as well as antimicrobial assay values (Kirby–Bauer disc diffusion and MIC measurements).

## 4. Conclusions

In the current research, to the best of our knowledge, 15 samples of propolis from across the whole Georgia state were characterized in detail by UHPLC-DAD-MS/MS for the first time. As expected, according to the chemical composition, they exhibited black poplar and aspen origin which result to be the main plant precursors of Georgian propolis. This plant origin was connected with high amounts of polyphenols in propolis, especially free phenolic acids, their monoesters, and glycerides as well as flavonoid aglycones.

This specific composition is connected with the strong antibacterial and antioxidant activities of Georgian propolis. Moreover, the dominance of black poplar resins over aspen may allow to expect high potential against staphylococci and other skin pathogens. The highest activity was found against different staphylococci strains and *C. albicans*, and confirmed using two different methods: the Kirby–Bauer disc diffusion method and by determination of MIC. For this reason, Georgian propolis may be an excellent raw material to prepare dermal drugs and cosmetics as well as sore throat remedies. On the other hand, the activity against other strains: *E. faecalis* 29212, *B. subtilis* 6633, *E. coli* 25922, *P. aeruginosa* 27853, and *K. pneumoniae* 700603, *C. krusei* 6258 was lower.

## Figures and Tables

**Table 1:** Chemical composition of 70% ethanolic extracts of Georgian propolis.

No.	Component	RT [min.]	UV Max [nm]	[M − H^+^]^−^	MS/MS Base Peak	MS/MS Secondary Peaks *m*/*z* (A [%])	Molecular Formula	Error [mDa]	Error [ppm]	RDB	RF
1	Gallic acid ^b^,^c^	2.2	**271**	169.0137	-	-	C_7_H_6_O_5_	0.6	3.4	5.0	-
2	4-Hydroxybenzoic acid ^a,b,c^	6.73	313, **282**	137.0243	137.0984	174.9986 (23.26), 159.2402 (5.08)	C_7_H_6_O_3_	0.1	0.8	5.0	[23,25,26,27]
3	Unidentified	9.89	-	165.0554	135.2359	93.1964 (66.58)	C_9_H_10_O_3_	0.3	1.7	5.0	-
4	Vanillin isomer ^b,c^	9.34	**310, 280**	151.0393	108.2066	-	C_8_H_8_O_3_	0.8	5.2	5.0	[23,25,27]
5	* 4-Hydroxybenzaldehyde ^b,c^	9.89	**282**	121.0293	-	-	C_7_H_6_O_2_	0.2	1.7	5.0	[23,27,28]
6	Caffeoylquinic acid	11.01	**324**	353.0875	191.1650	-	C_16_H_18_O_9_	0.4	1.0	8.0	[29]
7	Caffeic acid ^a,b,c^	11.56	**323**	179.0346	135.0449	107.0484 (8)	C_9_H_8_O_4_	0.4	2.0	6.0	[10,22,23,25]
8	Unidentified	12.3	**340**	369.0824	207.1554	192.1297 (85.93), 354.2545 (4.78)	C_16_H_18_O_10_	0.3	0.8	8.0	-
9	* Caffeoylglycerol ^b,c^	13.11	**320**	253.0711	161.0743	133.1839 (92.59), 135.1153 (40.05)	C_12_H_14_O_6_	0.6	2.5	6.0	[10,22,30]
10	* Pentenoyl hydroxyphenylpropionic acid isomer I ^c^	14.38	**320**	281.1036	161.1260	133.7160 (76.42)	C_14_H_18_O_6_	−0.5	−1.9	6.0	[31]
11	*p*-Coumaric acid ^a,b,c^	14.45	**310**	163.0401	119.1668	93.0893 (10.59)	C_9_H_8_O_3_	0.0	−0.1	6.0	[10,22,23,25]
12	* Pentenoyl hydroxyphenylpropionic acid isomer II ^b,c^	14.77	**320**	281.1034	161.1323	133.1404 (55.05), 135.1467 (28.11)	C_14_H_18_O_6_	−0.3	−1.0	6.0	[31]
13	Unidentified	14.87	**320**	267.0873	133.3121	160.1409 (56.58), 177.3359 (20.73), 252.2197 (15.82), 175.1448 (11.31), 105.1399 (7.69)	C_13_H_16_O_6_	0.1	0.3	6.0	[31]
14	Ferulic acid ^a,b,c^	15.24	**325**	193.0504	134.1169	-	C_10_H_10_O_4_	0.2	1.2	6.0	[10,22,23,25]
15	* Pentenoyl hydroxyphenylpropionic acid isomer III ^b,c^	15.26	**320**	281.1033	161.1496	133.2606 (51.72), 135.1486 (42.76), 179.1248 (10.81)	C_14_H_18_O_6_	−0.3	−1.0	6.0	[31]
16	Unidentified	15.54	**320**	147.0454	117.1541	-	C_9_H_10_O_2_	−0.2	−1.5	6.0	[31]
17	Isoferulic acid ^a,b,c^	15.75	**324**	193.0503	134.1466	-	C_10_H_10_O_4_	0.0	−0.2	6.0	[10,22,23,25]
18	Unidentified	16.09	-	375.1081	135.1110	161.1643 (95.36), 179.1848 (90.50), 153.2200 (11.83)	C_19_H_20_O_8_	0.4	1.2	10.0	-
19	Unidentified	16.28	**#370**	300.9988	301.1634	229.1050 (13.95), 284.2051 (14.24), 245.1424 (9.90), 201.1526 (7.75), 185.1520 (7.40), 257.1156 (6.16)	C_14_H_6_O_8_	0.2	0.8	12.0	-
20	* Caffeoylmalic acid (Phaseolic acid) isomer ^b,c^	16.64	**334**	295.0827	161.1286	133.2853 (58.51), 135.1555 (32.02)	C_14_H_16_O_7_	−0.3	−1.1	7.0	[23,32,33]
21	Unidentified	18.12	**#312**	359.1137	145.1441	119.1324 (55.67), 163.1822 (40.34), 117.2787 (26.02), 153.1744 (9.79), 150.2477 (5.81), 165.2462 (5.36)	C_19_H_20_O_7_	0.0	−0.1	10.0	-
22	Unidentified	18.53	**#312**	359.1141	145.1447	119.1192 (72.06), 163.2816 (58.22), 117.3281 (26.10), 153.1252 (9.74), 132.2316 (7.42)	C_19_H_20_O_7_	−0.4	−1.2	10.0	-
23	Ferulic acid derivate I ^b,c^	18.9	**#320**	389.1233	175.2187	134.2000 (97.52), 193.1598 (60.87), 160.1987 (65.05), 179.2051 (22.26), 162.8401 (12.90)	C_20_H_22_O_8_	0.9	2.2	10.0	[23]
24	Eriodictyol (4′-hydroxynaringenin) ^b,c^	18.94	**282**	287.0562	125.0569	177.1795 (70.51), 201.1649 (12.51), 259.2324 (12.01), 213.2079 (9.60), 241.2596 (8.96), 131.2132 (7.78)	C_15_H_12_O_6_	−0.1	−0.4	10.0	[23]
25	Ferulic acid derivate II ^b,c^	19.27	**#320**	389.1241	175.2185	134.1918 (99.08), 193.1674 (69.55), 160.4158 (63.35), 149.1159 (22.89), 153.1289 (11.69), 179.2038 (12.59), 195.2109 (6.85), 162.2295 (5.73), 117.1767 (4.60), 165.1998 (4.48), 151.1564 (4.31)	C_20_H_22_O_8_	0.1	0.3	10.0	[23]
26	Caffeic acid ethyl ester ^b,c^	19.52	**321**	207.0662	133.0306	135.0440 (62.42), 161.0241 (30.77), 106.0409 (3.87), 115.0228 (2.05)	C_11_H_12_O_4_	0.1	0.6	6.0	[25,31]
27	Unidentified	19.66	**#315**	279.0875	145.1378	117.1459 (61.44), 119.1462 (23.20)	C_14_H_16_O_6_	−0.1	−0.5	7.0	-
28	Unidentified	19.87	**#315**	279.0876	145.1427	117.2221 (48.55)	C_14_H_16_O_6_	−0.2	−0.6	7.0	-
29	Unidentified	20.56	**#320**	309.0980	133.3320	160.1377 (73.82), 175.1380 (31.14), 177.1554 (24.77), 294.1795 (17.22), 234.1876 (15.34)	C_15_H_18_O_7_	0.0	0.0	7.0	-
30	Unidentified	20.78	**#320**	309.0982	234.1821	160.1028 (88.05)	C_15_H_18_O_7_	−0.2	−0.7	7.0	-
31	Apigetrin ^b,c^	21.17	309, **265**	431.0983	268.2682	431.2804 (23.37), 240.1429 (9.85), 211.1568 (9.64)	C_21_H_20_O_10_	0.0	0.1	12.0	[10,22,23]
32	Unidentified	21.17	-	283.0610	196.2402	240.2042 (74.07), 268.1428 (63.61)	C_16_H_12_O_5_	0.2	0.7	11.0	-
33	^iw^ Cinnamic acid ^a,b,c^	21.36	**280**	-	-	-	-	-	-	-	[10,22,23,25]
34	Unidentified	21.77	**280**	285.0778	138.1476	224.1795 (91.95), 252.3284 (54.53), 239.2369 (42.40), 197.2624 (24.65)	C_16_H_14_O_5_	−1.0	−3.4	10.0	-
35	* Caffeic acid derivate ^c^	22.77	**#320**	207.0663	133.2670	135.1336 (46.89), 161.1030 (17.76)	C_11_H_12_O_4_	0.0	0.1	6.0	-
36	Unidentified	23.24	**308**	-	-	-	-	-	-	-	-
37	Pinobanksin 5-methylether ^b,c^	23.54	**287**	285.0777	252.0429	224.0470 (55.83), 138.0332 (38.07), 241.0481 (31.50), 165.0192 (14.95), 239.0674 (12.13), 195.0459 (12.02), 151.0027 (11.81), 213.0557 (11.34), 267.0660 (11.02), 285.0805 (9.31), 136.0190 (8.53), 107.0176 (6.81)	C_16_H_14_O_5_	−0.8	−2.9	10.0	[10,22,23,25]
38	* Caffeic acid derivate ^c^	24.35	**#320**	403.1393	179.1874	135.1179 (74.38), 161.1497 (53.58)	C_21_H_24_O_8_	0.5	1.3	10.0	-
39	di-Caffeoylglycerol ^b,c^	24.61	**320**	415.1033	253.2248	161.1173 (84.50), 179.1330 (65.63), 135.1406 (55.89)	C_21_H_20_O_9_	0.1	0.3	12.0	[30]
40	Quercetin ^a,b,c^	25.22	364, 270sh, **265**	301.0353	151.0034	121.0307 (29.41), 107.0140 (22.18), 149.0242 (14.01), 178.9969 (13.92), 301.0371 (7.58), 245.0461 (6.32), 273.0451 (5.48), 163.0034 (4.87), 211.0372 (3.84)	C_15_H_10_O_7_	0.1	0.3	11.0	[10,22,23,25]
41	* Flavonoid ^b,c^	25.52	**#370**	285.0412	133.1356	285.1812 (83.77), 151.0369 (33.21), 199.1521 (15.09), 107.1489 (12.83)	C_15_H_10_O_6_	−0.8	−2.7	11.0	-
42	Quercetin 3-methyl ether ^b,c^	27.02	355, 268sh, **255**	315.0497	271.0253	300.0274 (71.14), 255.0303 (42.89) 243.0297 (22.59), 227.0334 (2.55)	C_16_H_12_O_7_	0.2	0.5	11.0	[10,22,23,25]
43	Pinobanksin ^a,b,c^	27.45	**292**	271.0615	197.0617	253.0502 (89.28), 161.0604 (67.51), 271.0605 (56.26), 125.0242 (53.39), 151.0063 (30.14), 225.0558 (24.71), 107.0152 (23.97), 209.0588 (16.07), 185.0571 (15.86), 115.0559 (15.08), 157.0659 (14.43), 181.0651 (14.14), 215.0699 (11.83)	C_15_H_12_O_5_	−0.3	−1.1	10.0	[10,22,23,25]
44	* Diffractaic acid	28.21	-	357.1348	122.2211	342.2954 (33.31)	C_20_H_22_O_6_	−0.4	−1.1	10.0	[34]
45	Naringenin ^a,b,c^	28.80	**282**	271.0612	119.1344	151.0545 (43.37), 107.0883 (21.94), 187.2234 (10.00)	C_15_H_12_O_5_	0.0	0.1	10.0	[10,22,23,25]
46	Chrysin-5-methyl-ether ^b,c^	28.80	-	267.0662	224.1747	180.1680 (92.97), 252.1932 (26.27), 195.2896 (15.00)	C_16_H_12_O_4_	0.1	0.3	11.0	[10,22,23,25]
47	1-Caffeoyl-3-*p*-coumaroylglycerol ^b,c^	28.97	**312**	399.1085	163.1721	161.0857 (48.44), 119.1488 (48.96), 253.2139 (46.08), 179.1589 (25.62), 145.1790 (24.73), 235.1152 (20.40), 161.2192 (10.73), 237.2187 (8.31), 399.2525 (5.30)	C_21_H_20_O_8_	0	0.1	12.0	[10,22,23,30]
48	Unidentified	29.10	-	387.1451	145.1669	119.1386 (61.80), 163.2370 (64.64), 132.2356 (11.16), 195.1718 (7.30), 122.2278 (7.25)	C_21_H_24_O_7_	−0.2	−0.4	10.0	-
49	1-Caffeoyl-3-feruloylglycerol ^b,c^	29.45	323	429.119	193.1773	161.1135 (45.00), 253.2412 (47.74), 135.1340 (28.66), 179.1443 (20.67), 235.1809 (19.71), 175.1300 (18.00), 149.1476 (12.10), 429.3656 (8.98)	C_22_H_22_O_9_	0.1	0.2	12.0	[10,22,23,30]
50	Unidentified	29.69	**#282**	269.0822	150.0692	184.1621 (88.87), 165.1076 (80.74), 122.0565 (55.22), 254.1667 (50.90), 227.1995 (38.24), 269.26 (20.13)	C_16_H_14_O_4_	−0.3	−1	10.0	-
51	Unidentified	30.02	-	417.1560	175.1842	193.1740 (79.02), 134.1650 (64.70), 160.1591 (35.50), 178.4839 (34.02), 149.1311 (19.12), 195.1693 (9.00), 162.2682 (8.81), 203.2877 (6.49), 162.2628 (5.86), 312.2998 (4.03), 145.2405 (3.05)	C_22_H_26_O_8_	−0.5	−1.2	10.0	-
52	Apigenin ^a,b,c^	30.66	**338**, 290sh, 263	269.0457	117.0349	269.0455 (52.06), 151.0033 (39.01), 149.0245 (25.91), 227.0353 (12.66), 107.0138 (11.48), 225.0555 (10.59), 201.0561 (7.44), 183.0448 (6.40), 181.0630 (5.14), 121.0290 (4.92), 197.0608 (2.28)	C_15_H_10_O_5_	−0.2	−0.7	11.0	[10,22,23,25]
53	* Methylated flavonoid ^b,c^	30.79	-	299.0563	284.2118	137.0439 (29.24), 212.1925 (13.23), 228.1667 (9.72), 200.1613 (8.02), 186.3862 (7.94), 256.2071 (5.04), 214.2443 (2.23)	C_16_H_12_O_6_	−0.2	−0.7	11.0	-
54	Kaempferol ^a,b,c^	31.36	**366**, 295sh, 265	285.0405	285.0400	239.0335 (8.81), 187.0408 (8.20), 185.0580 (8.14), 229.0505 (7.99), 159.0464 (6.63)	C_15_H_10_O_6_	−0.1	−0.3	11.0	[10,22,23,25]
55	Unidentified	31.92	**310**	-	-	-	-	-	-	-	-
56	Quercetin-methyl-ether ^b,c^	31.96	-	315.0509	300.1989	151.1329 (26.66), 271.4108 (11.37), 164.1072 (7.61), 283.1502 (6.12), 148.0893 (5.64), 315.1957 (5.60), 255.2267 (4.65), 216.1788 (3.38), 108.2193 (2.95), 244.2404 (2.60), 136.2082 (2.55)	C_16_H_12_O_7_	0.1	0.3	11.0	[10,22,23,25]
57	Quercetin-methyl-ether ^b,c^	32.50	-	315.0511	300.1857	151.1387 (26.12), 271.2935 (11.15), 164.1172 (7.58), 283.1466 (5.81), 216.2658 (4.63)	C_16_H_12_O_7_	0.0	−0.1	11.0	[10,22,23,25]
58	Unidentified	32.67	-	387.1448	145.1580	163.2387 (72.09), 119.1315 (63.13), 132.2983 (10.57)	C_21_H_24_O_7_	0.1	0.3	10.0	-
59	Unidentified	32.93	-	259.1918	-	-	C_14_H_28_O_4_	−0.3	−1.1	1.0	-
60	(R/S) 1,2-di-*p*-Coumaroylglycerol isomer I ^b,c^	33.03	**312**, 300sh	383.1137	163.1661	119.1192 (71.11)	C_21_H_20_O_7_	−0.1	−0.3	12.0	[10,22,23,25]
61	Luteolin-5-methyl ether ^b,c^	33.21	350, 298sh, **267**	299.0549	255.0300	227.0344 (59.96), 284.0336 (15.07), 211.0379 (6.11)	C_16_H_12_O_6_	−0.2	−0.7	11.0	[10,22,23,25]
62	Unidentified	33.55	**320**	417.1558	193.1728	175.2081 (98.31), 134.1896 (74.55), 160.1626 (37.44), 149.1539 (23.02), 148.6488 (42.52), 179.1703 (18.22), 162.2589 (10.72), 162.2317 (8.37), 149.7628 (8.20)	C_22_H_26_O_8_	−0.3	−0.7	10.0	-
63	Quercetin-di-methyl-ether ^b,c^	33.91	256, 354	329.0669	271.1688	299.1957 (99.34), 243.1827 (90.63), 285.4120 (51.12), 257.2245 (31.51), 314.2443 (29.44), 227.1660 (5.23), 215.1776 (3.74), 199.1937 (3.06), 255.1517 (2.88)	C_17_H_14_O_7_	−0.2	−0.6	11.0	[10,22,23,25]
64	1,3-di-*p*-Coumaroylglycerol ^b,c^	33.98	**312**	383.1143	163.1491	119.1294 (69.49), 145.1419 (61.09), 117.2337 (8.68), 219.1918 (7.20), 237.1927 (6.59), 383.3604 (2.42)	C_21_H_20_O_7_	−0.7	−1.8	12.0	[10,22,23,25]
65	(R/S) 1-*p*-Coumaroyl-3-feruloylglycerol ^b,c^	34.48	**316**	413.1241	193.1678	163.1401 (97.02), 134.1556 (76.61), 119.1270 (54.22), 145.1831 (49.19), 175.1423 (37.15), 149.1613 (18.59), 398.3044 (15.16), 161.2714 (11.03), 413.4833 (10.86), 219.2266 (8.25), 237.2114 (7.99), 249.2240 (7.20), 252.2234 (6.36), 267.1968 (5.71), 235.2153 (5.19)	C_22_H_22_O_8_	0.1	0.2	12.0	[10,22,23,25]
66	Galangin-5-methyl-ether ^b,c^	34.58	**353**	283.0612	211.1796	239.2387 (58.94), 283.2956 (5.07), 268.1859 (4.79)	C_16_H_12_O_5_	0.0	−0.1	11.0	[10,22,23,25]
67	(R/S) 1,2-di-*p*-Coumaroylglycerol isomer II ^b,c^	34.7	**315**	383.1137	163.1447	119.1053 (78.80), 145.1222 (70.92)	C_21_H_20_O_7_	−0.1	−0.2	12.0	[10,22,23,25]
68	5-Methyl-pinobanksin-3- acetate ^b,c^	34.69	**280**	327.0878	224.1781	267.2163 (67.46), 252.1858 (62.85), 285.2285 (45.11), 239.5247 (36.67)	C_18_H_16_O_6_	−0.4	−1.1	11.0	[10,22]
69	1,3-di-Feruloylglycerol ^b,c^	34.84	**320**	443.1348	193.1648	134.1517 (58.91), 175.1561 (37.23), 149.1415 (19.74), 428.3535 (15.30), 160.4916 (15.61), 249.2107 (10.66), 207.2209 (7.82), 443.3781 (8.72), 267.2599 (6.21), 235.2054 (5.53)	C_23_H_24_O_9_	0.0	−0.1	12.0	[10,22,23,30]
70	2-Acetyl-1,3-di-caffeoylglycerol ^b,c^	35.25	**320**	457.1141	179.1565	161.1483 (77.42), 135.1105 (45.90), 235.2026 (48.11), 295.2730 (38.65), 457.3254 (5.86), 173.1999 (3.85), 397.3589 (4.20), 413.5593 (3.26), 253.2546 (2.22)	C_23_H_22_O_10_	−0.1	−0.2	13.0	[22,23,25,30]
71	Quercetin-methyl-ether ^b,c^	36.81	**362**	315.0509	165.1079	121.1282 (39.04), 300.2162 (27.72), 151.1032 (9.49), 272.2119 (6.69), 244.2122 (4.72), 256.2717 (3.45)	C_16_H_12_O_7_	0.1	0.4	11.0	[10,22,23,25]
72	Kaempferol-methyl-ether ^b,c^	36.90	-	299.0563	284.1907	299.2151 (7.35), 256.1440 (5.21), 133.2419 (5.23), 151.0642 (2.37), 227.3301 (2.53)	C_16_H_12_O_6_	−0.2	−0.7	11.0	[31]
73	Caffeic acid butyl or isobutyl ester isomer isomer I ^b,c^	37.46		235.0978	133.5359	161.1498 (41.79)	C_13_H_16_O_4_	−0.2	−1	6.0	[10,22,23,25]
74	Pinobanksin-3-*O*-hydroxybutyrate or isobutyrate ^b,c^	37.82	**278**	357.0975	253.2301	271.2704 (5.41), 197.1954 (4.82)	C_19_H_18_O_7_	0.5	1.3	11.0	
75	Caffeic acid butyl or isobutyl ester isomer II ^b,c^	38.22		235.0976	161.1424	135.1301 (93.59)	C_13_H_16_O_4_	−0.1	−0.2	6.0	[10,22,23,25]
76	* Caffeic acid prenyl ester isomer ^b,c^	38.15	**320**	247.0975	135.1279	161.1137 (33.38)	C_14_H_16_O_4_	0.0	0.2	7.0	[10,22,23,25]
77	* Flavonoid ^b,c^	38.66	-	313.0719	283.2034	255.1726 (88.05), 298.2100 (43.79), 269.1878 (35.50)	C_17_H_14_O_6_	−0.1	−0.3	11.0	-
78	Quercetin-dimethyl-ether ^b,c^	39.30	**353**	329.0669	299.1970	271.1734 (30.28), 314.2379 (21.06), 285.2543 (2.46)	C_17_H_14_O_7_	−0.3	−0.8	11.0	[10,22,23,25]
79	Caffeic acid 2-methyl-2-butenyl ester ^b,c^	39.50	**325**	247.0979	135.1258	161.1463 (36.02), 179.1152 (11.25)	C_14_H_16_O_4_	−0.4	−1.5	7.0	[10,22,23,25]
80	Caffeic acid derivate ^b,c^	40.56	-	269.0817	134.1571	161.1133 (29.48)	C_16_H_14_O_4_	0.2	0.9	10.0	-
81	Caffeic acid 3-methyl-2-butenyl ester (Basic prenyl ester) ^b,c^	40.91	**325**	247.0979	134.2235	106.1200 (6.32)	C_14_H_16_O_4_	−0.4	−1.7	7.0	[10,22,23,25]
82	Caffeic acid 3-methyl-3-butenyl ester ^b,c^	41.42	**325**	247.0977	134.2234	106.1659 (5.64)	C_14_H_16_O_4_	−0.1	−0.4	7.0	[10,22,23,25]
83	(*R*/*S*) 2-Acetyl-1-caffeoyl-3-*p*-coumaroylglycerol ^b,c^	41.91	**315**	441.1197	163.1479	179.1479 (85.75), 161.1248 (42.10), 135.1226 (40.85), 145.1602 (39.56), 119.1276 (35.73), 235.2124 (27.59), 295.2823 (14.64), 219.1731 (7.31), 173.1816 (6.88), 381.3956 (7.79), 217.1798 (4.50), 441.3513 (4.75), 189.1920 (3.80), 277.2596 (2.86)	C_23_H_22_O_9_	−0.6	−1.3	13.0	[10,23,25,30]
84	Chrysin ^a,b,c^	42.38	312sh, **268**	253.0505	253.0507	143.0507 (41.53), 145.0299 (21.10), 209.0611 (14.10), 107.0142 (13.33), 181.0652 (8.16), 185.0615 (6.19)	C_15_H_10_O_4_	−0.7	−2.8	11.0	[10,22,23,25]
85	Caffeic acid benzyl ester ^b,c^	42.69	**326**	269.0818	134.1302	161.0235 (22.96), 137.0256 (4.03)	C_16_H_14_O_4_	−0.3	−1.1	10.0	[10,22,23,25]
86	(*R*/*S*) 2-Acetyl-1-caffeoyl-3-feruloylglycerol ^b,c^	42.71	**325**	471.1297	193.1684	179.1426 (89.35), 161.1376 (39.08), 135.1206 (36.34), 175.1354 (30.55), 235.2142 (27.00), 295.2633 (15.17), 149.1373 (11.76), 411.3719 (10.46), 173.2002 (6.78), 471.4677 (7.40), 249.2085 (5.71), 217.2027 (5.85), 189.2351 (3.58), 277.2277 (3.10), 367.3075 (2.44)	C_24_H_24_O_10_	−0.1	−0.1	13.0	[10,22,23,25]
87	* Sakuranetin isomer ^c^	43.29	**287**	285.0769	119.1310	165.1100 (17.55), 150.1056 (7.14), 121.1330 (4.34)	C_16_H_14_O_5_	0.0	−0.1	10.0	-
88	Pinocembrin ^b,c^	43.41	**290**	255.0666	171.0464	151.0040 (80.69), 255.0662 (75.17), 213.0557 (74.89), 145.0662 (70.09), 107.0148 (52.59), 185.0609 (34.69), 169.0660 (24.91), 211.0753 (23.68), 164.0102 (17.93), 187.0757 (16.78), 136.0166 (16.34)	C_15_H_12_O_4_	−0.2	−0.8	10.0	[10,22,23,25]
89	(*R*/*S*) 1-Acetyl-2-caffeoyl-3-feruloylglycerol	43.63	**320**	471.1298	193.1467	179.1396 (85.32), 135.1399 (37.94), 161.1220 (37.43), 175.1639 (31.33), 235.1730 (27.79), 295.2133 (17.68)	C_24_H_24_O_10_	−0.1	−0.3	13.0	[10,22,23,25]
90	Sakuranetin ^b,c^	44.69	**290**	285.0773	124.1060	139.1376 (64.17), 145.1010 (42.28), 148.0978 (8.73), 165.1128 (4.71)	C_16_H_14_O_5_	−0.4	−1.6	10.0	[10,22,23,25]
91	Galangin ^a,b,c^	45.17	360, **266**	269.0454	269.0454	169.0659 (12.64), 171.0448 (10.87), 213.0554 (10.73), 143.0502 (8.90), 223.0421 (8.03,) 195.0463 (7.34)	C_15_H_10_O_5_	−0.2	−0.8	11.0	[10,22,23,25]
92	Acacetin ^a,b,c^	45.78	335, **269**	283.0614	268.1865	240.1463 (6.26), 117.1239 (5.07), 283.3149 (4.20), 151.0439 (2.69)	C_16_H_12_O_5_	−0.2	−0.8	11.0	[10,22,23,25]
93	Ermanin isomer ^b,c^	46.13	333, **275**	313.0721	283.1860	298.2345 (15.38), 255.1818 (14.54), 163.0741 (7.37), 227.1523 (3.56), 117.1008 (2.59), 165.2551 (3.29)	C_17_H_14_O_6_	−0.3	−1.1	11.0	[22,23,25,27]
94	Caffeic acid pentyl or isopentylester ^b,c^	46.82	-	249.1138	161.1050	-	C_14_H_18_O_4_	−0.6	−2.3	6.0	[10,22,23]
95	Caffeic acid phenethyl ester (CAPE) ^b,c^	47.21	326	283.0981	135.1231	161.1478 (46.24), 179.1445 (20.40)	C_17_H_16_O_4_	−0.6	−2.0	10.0	[10,22,23,25]
96	Pinobanksin 3-*O*-acetate ^b,c^	47.69	**295**	313.0725	253.051	197.0611 (5.86), 271.0616 (5.36), 209.0610 (4.75), 143.0503 (3.17)	C_17_H_14_O_6_	−0.7	−2.3	16.0	[10,22,23,25]
97	Kaempferide (Kaempferol 4’-methyl ether) ^b,c^	47.73	**365**, 267	299.0563	284.2046	151.0766 (31.84), 164.0964 (10.53), 107.1859 (6.32), 132.1238 (4.91), 228.1712 (3.34), 299.2162 (3.46), 200.1766 (2.10), 256.1541 (2.02)	C_16_H_12_O_6_	−0.2	−0.7	11.0	[10,22,23,25]
98	Methoxychrysin ^b,c^	48.06	310sh, **266**	269.0447	211.1827	239.1608 (34.16), 269.1189 (18.83)	C_15_H_10_O_5_	0.8	3.1	11.0	[10,22,23,25]
99	Quercetin-dimethyl ether ^b,c^	48.19	**#370**	329.0667	271.1883	299.1853 (14.42), 314.2161 (4.04), 243.1375 (2.85)	C_17_H_14_O_7_	0.0	0.0	11.0	[10,22,23,25]
100	Ermanin (Kaempferol-3,4′-dimethyleter) ^b,c^	30.65	**350**, 267	313.0719	283.2122	255.1799 (24.32), 253.1653 (17.11), 298.2169 (10.64)	C_17_H_14_O_6_	−0.1	−0.3	11.0	[22,23,25,27]
101	*p*-Coumaric acid 3-methyl-3-butenyl ester ^b,c^	50.69	**313**	231.1028	117.1725	119.1277 (90.59), 145.1345 (49.02), 163.1427 (4.99)	C_14_H_16_O_3_	−0.1	−0.4	7.0	[10,22,23,25]
102	2-Acetyl-1,3-di-*p*-coumaroylglycerol ^b,c^	50.93	**312**	425.1242	163.0403	145.0296 (53.67), 119.0502 (49.02), 219.0658 (11.88), 215.0706 (6.36), 237.0917 (5.21), 171.0817 (5.05), 117.0364 (4.31)	C_23_H_22_O_8_	0.0	0.1	13.0	[10,22,23,25]
103	Ayanin (3,7,4′-trimethylquercetin) ^b,c^	51.40	271, **334**	343.0822	270.1821	285.2314 (81.68), 313.2589 (62.40), 328.2901 (28.51), 298.2102 (20.86)	C_18_H_16_O_7_	0.2	0.5	11.0	[23]
104	(*R*/*S*) 2-Acetyl-3-*p*-coumaroyl-1-feruloylglycerol ^b,c^	51.87	**316**	455.1336	163.1189	193.1641 (95.43), 134.1510 (43.39), 119.1319 (41.07), 145.1470 (38.25), 175.3908 (43.52), 160.7224 (15.25)	C_24_H_24_O_9_	1.1	2.5	13.0	[10,22,23,25]
105	(*R*/*S*) 1-Acetyl-2,3-di-*p*-coumaroylglycerol ^b,c^	51.98	**311**	425.1244	163.1361	145.1342 (64.46), 119.1378 (57.20), 219.2043 (13.02), 171.4749 (7.70)	C_23_H_22_O_8_	−0.2	−0.4	13.0	[10,22,23,25]
106	*p*-Coumaric acid 3-methyl-2-butenyl or 2-methyl-2-butenyl ester ^b,c^	52.18	**313**	231.1027	117.2347	-	C_14_H_16_O_3_	0.0	0.0	7.0	[10,22,23,25]
107	2-Acetyl-1,3-di-feruloylglycerol ^b,c^	52.49	**324**	485.1456	193.1733	175.1362 (33.53), 134.1327 (31.79), 149.1651 (12.96), 249.2397 (8.24), 230.3454 (7.88), 160.3150 (7.78), 425.4171 (4.94), 207.1350 (4.01), 470.4230 (4.63)	C_25_H_26_O_10_	−0.3	−0.5	13.0	[10,22,23,25]
108	(R/S) 2-Acetyl-3-*p*-coumaroyl-1-feruloylglycerol ^b,c^	52.79	**311**	455.1345	163.147	193.1701 (88.76), 145.1835 (50.56), 134.1724 (48.23), 119.1170 (43.62), 175.3530 (48.29), 149.1473 (15.69), 219.1762 (12.00), 160.6531 (16.12), 249.1829 (10.51), 230.2269 (10.13), 215.4859 (6.59), 234.2500 (4.96)	C_24_H_24_O_9_	0.2	0.5	13.0	[10,22,23,25]
109	(R/S) 1-Acetyl-2-*p*-coumaroyl-3-feruloylglycerol ^b,c^	53.01	**315**	455.1347	163.1173	193.1616 (78.06), 134.1637 (46.98), 145.0907 (41.86), 175.1441 (42.27), 119.1468 (40.73)	C_24_H_24_O_9_	0.1	0.2	13.0	[10,22,23,25]
110	Unidentified	53.54	-	311.2229	157.1776	153.2286 (38.11)	C_18_H_32_O_4_	−0.1	−0.2	3.0	-
111	*p*-Coumaric acid benzyl ester ^b,c^	53.88	**316**	253.0869	117.2666	145.1076 (12.89), 121.3249 (3.15)	C_16_H_14_O_3_	0.1	0.3	10.0	[10,22,23,25]
112	(*R*/*S*) 1-Acetyl-2,3-di-feruloylglycerol ^b,c^	53.9	**324**	485.1455	193.1715	134.1509 (38.23), 175.1523 (36.54), 149.1409 (13.00), 160.2415 (9.74), 249.2341 (8.80), 230.4313 (9.02)	C_25_H_26_O_10_	−0.2	−0.4	13.0	[10,22,23,25]
113	Unidentified	54.17	-	295.0978	134.1210	-	C_18_H_16_O_4_	−0.2	−0.7	11.0	-
114	Unidentified	55.03	**305**	433.0927	243.2176	271.2540 (40.28), 415.3610 (25.86), 161.1105 (21.48), 253.2210 (10.71), 125.1055 (7.37), 135.1193 (6.47), 165.1139 (5.55), 152.0896 (5.35), 180.0904 (4.98), 227.2045 (4.58), 199.2596 (4.10), 371.2968 (3.52), 280.2369 (2.60)	C_24_H_18_O_8_	0.2	0.4	16.0	-
115	^iw^ Ferulic acid benzyl ester ^b,c^	55.35	**320**	283.0975	133.1109	160.2162 (13.55)	C_17_H_16_O_4_	0.1	0.4	10.0	[10,22,23,25]
116	Caffeic acid cinnamyl ester ^b,c^	56.10	**323**	295.0982	134.1352	161.1277 (5.53), 137.1107 (5.18), 106.1119 (4.21)	C_18_H_16_O_4_	−0.6	−1.9	11.0	[10,22,23,25]
117	Pinobanksin-3-*O*-propanoate ^b,c^	58.20	**294**	327.0878	253.2179	197.2305 (5.41), 209.2052 (3.72), 271.2717 (2.71), 143.1575 (2.09)	C_18_H_16_O_6_	−0.4	−1.2	11.0	[10,22,23,25]
118	*p*-Coumaric acid phenethyl ester ^b,c^	58.46	**310**	267.1031	119.1219	145.1261 (81.97), 117.2176 (80.24), 163.1240 (11.83)	C_17_H_16_O_3_	−0.4	−1.6	10.0	[10,22,23,25]
119	Pinostrobin chalcone ^b,c^	60.56	**343**	269.0827	122.0703	165.1175 (83.49), 253.4170 (86.88), 177.1620 (49.29), 226.2073 (47.58), 171.1475 (35.51), 150.0776 (31.31), 163.0634 (21.30), 269.2267 (16.42), 136.1084 (13.47), 198.2301 (14.25)	C_16_H_14_O_4_	−0.3	−0.8	10.0	[22,23]
120	* Flavonoid	62.12	**280**	271.0979	152.0937	124.0742 (60.13), 210.2039 (27.77), 238.2594 (25.34), 173.1662 (13.05), 165.1188 (10.13), 271.2509 (7.97), 253.2077 (6.31)	C_16_H_16_O_4_	−0.3	−1.1	9.0	-
121	^iw^ Tectochrysin	63.00	313, **268**	-	-	-	-	-	-	-	[22]
122	^iw^ Pinostrobin	63.48	**288**	-	-	-	-	-	-	-	[22,23]
123	*p*-Coumaric acid cinnamyl ester ^b,c^	64.11	**313**	279.1029	117.3253	-	C_18_H_16_O_3_	−0.3	−1.0	11.0	[10,23,25,27]
124	Unidentified	64.24	-	321.2439	321.4590	-	C_20_H_34_O_3_	−0.4	−1.3	4.0	-
125	Unidentified	64.41	-	521.2767	259.3499	163.1301 (29.97), 145.1084 (23.24), 521.6452 (30.79), 321.3004 (23.05), 219.1630 (15.02), 241.2996 (11.53), 461.5731 (12.76), 261.2290 (11.09), 503.6358 (11.84), 279.2142 (6.74), 443.5010 (4.40)	C_28_H_42_O_9_	−1.1	−1.2	8.0	-
126	Unidentified	64.78	-	551.2874	259.3522	551.6766 (46.94), 193.1367 (23.75), 175.1355 (17.53), 491.4771 (12.12), 351.3076 (11.71), 249.1760 (8.86), 291.2003 (7.55), 533.5316 (7.72), 536.5437 (6.33), 309.3566 (5.23)	C_29_H_44_O_10_	−1.1	−1.2	8.0	-
127	Unidentified	64.6	**323**	-	-	-	-	-	-	-	
128	Pinobanksin 3-*O*-butanoate or isobutanoate ^b,c^	64.92	**293**	341.1037	253.2173	197.2078 (4.89), 209.1812 (3.17)	C_19_H_18_O_6_	−0.6	−1.8	11.0	[10,23,25,27]
129	Pinobanksin 3-*O*-pentenoate or isopentenoate isomer I ^b,c^	65.55	**292**	353.1039	253.2231	197.2305 (4.88), 209.1898 (2.96)	C_20_H_18_O_6_	−0.9	−2.5	12.0	[10,23,25,27]
130	Pinobanksin 3-*O*-pentenoate or isopentenoate isomer II ^b,c^	65.90	**282**	353.1035	253.2266	271.2152 (26.83), 197.2792 (5.55), 209.5579 (3.51), 225.2615 (2.59)	C_20_H_18_O_6_	−0.5	−1.9	12.0	[10,23,25,27]
131	Pinobanksin 3-*O*-benzoate ^b,c^	66.91	**#278**	375.0878	253.2202	197.1308 (4.84), 225.1950 (3.56), 121.1922 (3.04), 209.1906 (2.85)	C_22_H_16_O_6_	−0.4	−1.0	15.0	[31]
132	Unidentified	67.34	**279**	-	-	-	-	-	-	-	-
133	Unidentified	67.77	-	519.3697	473.7448	373.5722 (30.52), 471.6560 (27.01), 385.5068 (5.37)	C_31_H_52_O_6_	−0.6	−1.2	6.0	-
134	Pinobanksin 3-*O*-pentanoate or isopentenoate isomer I ^b,c^	67.88	**293**	355.1192	253.2167	197.2052 (4.62), 271.2241 (3.55), 209.1801 (2.17)	C_20_H_20_O_6_	−0.5	−1.5	11.0	[10,22,23,25]
135	Pinobanksin 3-*O*-pentanoate or isopentenoate isomer II ^b,c^	68.02	**293**	355.1194	253.2180	197.2292 (4.47), 209.1992 (2.52)	C_20_H_20_O_6_	−0.6	−1.8	11.0	[10,22,23,25]
136	Unidentified	68.18	-	315.1606	134.2110	137.0773 (4.72), 179.1280 (2.29)	C_19_H_24_O_4_	−0.4	−1.3	8.0	-
137	Unidentified	68.23	-	463.3284	283.4493	-	C_24_H_48_O_8_	−0.8	−1.7	1.0	-
138	Pinobanksin 3-*O*-hexenoate or isohexenoate ^c^	68.64	-	367.1189	253.2181	271.2341 (31.89), 197.2592 (5.77), 209.4797 (3.20), 225.2691 (2.91)	C_21_H_20_O_6_	−0.2	−0.4	12.0	-
139	Unidentified	68.86	-	471.3479	471.6653	-	C_30_H_48_O_4_	0.1	0.3	7.0	-
140	Pinobanksin-3-*O*-cinnamate ^c^	69.00	**278**	401.1033	253.2046	197.1602 (4.77), 225.2060 (2.94)	C_24_H_18_O_6_	−0.2	−0.6	16.0	-
141	Pinobanksin-3-*O*-hydroxycinnamate^,c^	69.31	**285**	403.1197	253.2276	271.2222 (4.98), 197.2242 (4.05), 225.3038 (2.92), 149.1545 (2.44)	C_24_H_20_O_6_	−1.0	−2.5	15.0	[31]
142	Metoxycinnamic acid cinnamyl ester ^b,c^	69.35	**282**	293.2125	293.4701	185.1883 (57.87), 125.1730 (49.45), 141.2221 (18.74), 197.3495 (15.90), 97.2334 (11.61)	C_18_H_30_O_3_	−0.3	−0.9	4.0	[10,22,23,25]
143	Unidentified	69.66	-	531.3696	489.6876	531.7291 (51.67), 389.4929 (25.91), 471.6462 (26.77), 371.4896 (3.21), 431.5416 (2.59)	C_32_H_52_O_6_	−0.4	−0.8	7.0	-
144	Pinobanksin 3-*O*-hexanoate or isohexanoate isomer I ^b,c^	69.67	**281**	369.1347	253.2138	271.2252 (4.95), 197.1623 (3.43), 225.1455 (2.37), 115.1797 (1.95)	C_21_H_22_O_6_	−0.3	−0.8	11.0	[10,22,23,25]
145	Unidentified	69.80	-	473.3641	473.6798	373.5787 (6.14)	C_30_H_50_O_4_	−0.5	−1.1	6.0	
146	Pinobanksin 3-*O*-hexanoate or isohexanoate isomer II ^b,c^	69.96	**281**	369.1347	253.2245	197.2037 (4.52), 271.2081 (3.90), 225.2958 (2.22), 209.1639 (1.98), 115.1717 (1.93)	C_21_H_22_O_6_	−0.3	−0.8	11.0	[10,22,23,25]
147	Unidentified	70.2	-	533.3855	533.7199	491.6832 (47.24), 473.6830 (25.96)	C_32_H_54_O_6_	−0.8	−1.4	6.0	-
148	Unidentified	70.34	-	343.2855	283.3972	211.3522 (96.37), 197.2944 (72.36), 253.4190 (30.83), 279.4765 (19.71)	C_20_H_40_O_4_	−0.1	−0.3	1.0	-
149	Unidentified	70.73	-	295.2279	295.4866	141.2001 (52.92)	C_18_H_32_O_3_	0.0	−0.1	3.0	-
150	Pinobanksin 3-*O*-phenylpentenoate or phenyl isopentenoate ester^,c^	70.97	**#282**	429.1344	253.2249	271.2379 (57.79), 197.1788 (3.17), 225.3905 (3.81)	C_26_H_22_O_6_	0.0	−0.1	16.0	-
151	Unidentified	71.18	-	469.3316	469.648	-	C_30_H_46_O_4_	0.7	1.5	8.0	-

Table legend: No—number; UV max [nm]—maximum of UV absorption, higher maximum is bolded; RBD—ring and double bond equivalents; - component did not produce ion or did not have UV spectrum (or too low concentration); #—UV spectrum is weak due to low concentration and its maximum is unclear; ^a^ component identified by comparison with standard; ^b^ component identified by comparison with literature; ^c^ component identified by prediction of mass fragment and UV spectrum; * component tentatively identified; ^iw^ component does not produces or produce low/trace amount of ions in negative mode.

**Table 2:** Presence of components in UHPLC-DAD-MS/MS profile of 70% ethanolic extracts of Georgian propolis.

No.	Component	RT MS	UV Max [nm]	[M − H^+^]^−^	ASP	NOR	PAS	MES	ORG	VAR	OTA	QVA	U.R.1	U.R.1	MTS	KAK	AKH	DUS	IME
1	Gallic acid ^b,c^	2.2	**271**	169.0137	-	-	-	+	-	-	-	-	-	-	-	-	-	-	-
2	4-Hydroxybenzoic acid ^a,b,c^	6.73	313, **282**	137.0243	+	+	+	tr	+	+	+	+	+	+	+	+	+	+	+
3	Unidentified	9.89	-	165.0554	-	-	-	+	-	-	-	+	-	-	-	-	-	-	-
4	Vanillin isomer ^b,c^	9.34	310, 280	151.0393	-	-	-	-	-	-	-	+	-	-	-	-	-	-	-
5	* 4-Hydroxybenzaldehyde ^b,c^	9.89	**282**	121.0293	-	tr	tr	+	-	-	-	-	-	-	-	-	-	-	-
6	Caffeoylquinic acid	11.01	**324**	353.0875	-	-	-	-	-	-	-	-	+	+	++	-	-	-	-
7	Caffeic acid ^a,b,c^	11.56	**323**	179.0346	++	+++	++	+	+++	+++	+++	+++	+++	+++	+++	++	++	+++	++
8	Unidentified	12.3	**340**	369.0824	-	-	-	-	-	-	-	-	+	+	-	-	-	-	-
9	* Caffeoylglycerol ^b,c^	13.11	**320**	253.0711	+	+	++	+++	-	+	+	+	+	+	+	-	+	-	+
10	* Pentenoyl hydroxyphenylpropionic acid isomer I ^b,c^	14.38	**320**	281.1036	+	+	-	-	+	-	+	+	+	+	-	tr	tr	tr	-
11	*p*-Coumaric acid ^a,b,c^	14.45	**310**	163.0401	++	+++	+++	+	+++	+++	+++	+++	+++	+++	+++	++	++	+++	++
12	* Pentenoyl hydroxyphenylpropionic acid isomer II ^b,c^	14.77	**320**	281.1034	+	+	tr	-	+	+	+	+	+	+	+	tr	tr	tr	tr
13	Unidentified	14.87	**320**	267.0873	-	tr	+	+	-	-			-	-	-	-	-	-	-
14	Ferulic acid ^a,b,c^	15.24	**325**	193.0504	+	++	+++	+++	+	+	-	+	++	++	+	+	+	+	-
15	* Pentenoyl hydroxyphenylpropionic acid isomer III ^b,c^	15.26	**320**	281.1033	+	tr	tr	-	+	+	+	tr	tr	tr	+	tr	tr	tr	tr
16	Unidentified	15.54	**320**	147.0454	-	-	+	+	-	-	-		-	-	+	-	-	-	-
17	Isoferulic ^a,b,c^	15.75	**324**	193.0503	++	+++	++	-	+++	+++	+++	+++	+++	+++	+++	++	++	+++	++
18	Unidentified	16.09	-	375.1081	-	-	tr	+	-	-	-	-	-	-	-	-	-	-	-
19	Unidentified	16.28	**#370**	300.9988	-	-	tr	+	-	-	-	-	-	-	-	-	-	-	-
20	* Caffeoylmalic acid (Phaseolic acid) isomer ^b,c^	16.64	**334**	295.0827	-	+	tr	+	+	-	-	+	-	-	-	-	-	-	tr
21	Unidentified	18.12	**#312**	359.1137	-	-	tr	+	-	-	-	-	-	-	-	-	-	-	-
22	Unidentified	18.53	**#312**	359.1141	-	-	+	+	-	-	-	-	-	-	-	-	-	-	-
23	Ferulic acid derivate I ^b,c^	18.90	**#320**	389.1233	-	-	tr	+	-	-	-	-	-	-	-	-	-	-	-
24	Eriodictyol (4′-hydroxynaringenin) ^b,c^	18.94	**282**	287.0562	+	+	tr	+	+	+	+	+	+	+	tr	tr	tr	tr	tr
25	Ferulic acid derivate II ^b,c^	19.27	**#320**	389.1241	-	-	+	+	-	-	-	-	-	-	-	-	-	-	-
26	Caffeic acid ethyl ester ^b,c^	19.52	**321**	207.0662	+	++	++	-	++	++	++	++	++	++	++	+	+	++	++
27	Unidentified	19.66	**#315**	279.0875	-	-	+	+	-	-	-	+	-	-	-	-	-	-	-
28	Unidentified	19.87	**#315**	279.0876	-	+	+	+	-	-	-	-	-	-	-	-	-	-	-
29	Unidentified	20.56	**#320**	309.098	-	-	tr	+	-	-	-	-	-	-	-	-	-	-	-
30	Unidentified	20.78	**#320**	309.0982	-	-	tr	+	-	-	-	-	-	-	-	-	-	-	-
31	Apigetrin ^b,c^	21.17	309, **265**	431.0983	+	-	-	-	+	+	+	tr	+	+	tr	tr	+	tr	tr
32	Unidentified	21.17	-	283.061	+	-	-	-	-	-	-	-	-	-	-	-	-	-	
33	^iw^ Cinnamic acid ^a,b,c^	21.36	**280**	-	-	++	-	-	-	+	+	-	+	+	-	-	-	-	
34	Unidentified	21.77	**280**	285.0778	+	-	-	-	+	-	-	-	+	+	-	-	+	-	tr
35	* Caffeic acid derivate ^c^	22.77	**#320**	207.0663	-	+	-	-	tr	-	-	tr	-	-	-	-	-	-	
36	Unidentified	23.24	**308**	-	+	++	+	-	++	+	+	++	+	+	-	++	-	++	
37	Pinobanksin 5-methylether ^b,c^	23.54	**287**	285.0777	++	+	+	-	++	+	++	+	++	++	+++	+++	+++	+++	++
38	* Caffeic acid derivate ^c^	24.35	**#320**	403.1393	-	-	-	+	-	-	-	-	-	-	-	-	-	-	-
39	di-Caffeoylglycerol ^b,c^	24.61	**320**	415.1033	-	-	-	+	-	-	-	-	-	-	-	-	-	-	-
40	Quercetin ^a,b,c^	25.22	364, 270sh, **265**	301.0353	+	tr	tr	+	+	+	+	+	+	+	tr	+	+	+	+
41	* Flavonoid ^b,c^	25.52	**#370**	285.0412	+	tr	-	-	+	+	+	+	+	+	tr	tr	+	tr	-
42	Quercetin 3-methyl ether ^b,c^	27.02	355, 268sh, **255**	315.0497	+	+	tr	-	+	+	+	+	+	+	+	+	+	+	+
43	Pinobanksin ^a,b,c^	27.45	**292**	271.0615	+++	++	+	-	+++	+++	+++	++	+++	+++	+++	+++	+++	+++	+++
44	* Diffractaic acid (uncertain)	28.21	-	357.1348	+	-	-	-	-	-	-	-	-	-	-	-	-	-	-
45	Naringenin ^a,b,c^	28.80	**282**	271.0612	+	tr	+	+	+	+	+	+	+	+	+	+	+	+	+
46	Chrysin-5-methyl-ether ^b,c^	28.80	-	267.0662	+	-	-	-	-	+	-	-	-	-	-	-	-	-	-
47	1-Caffeoyl-3-*p*-coumaroylglycerol ^b,c^	28.97	**312**	399.1085	-	tr	tr	+	-	-	-	-	-	-	-	-	-	-	-
48	Unidentified	29.10	-	387.1451	-	-	tr	+	-	-	-	-	-	-	-	-	-	-	-
49	1-Caffeoyl-3-feruloylglycerol ^b,c^	29.45	323	429.1190	-	-	tr	+	-	-	-	-	-	-	-	-	-	-	-
50	Unidentified	29.69	**#282**	269.0822	+	tr	-	-	+	+	+	tr	tr	tr	-	tr	+	tr	tr
51	Unidentified	30.02	-	417.1560	-	-	tr	+	-	-		-	-	-	-	-	-	-	-
52	Apigenin ^a,b,c^	30.66	**338**, 290sh, 263	269.0457	+	+	+	+	++	+	+	++	+	+	++	++	+	++	++
53	* Methylated flavonoid ^b,c^	30.79	-	299.0563	-	+	+	+	tr	-	-	-	tr	tr	-	-	-	-	-
54	Kaempferol ^a,b,c^	31.36	**366**, 295sh, 265	285.0405	+	+	+	+	+	+	+	+	+	+	+	+	+	+	+
55	Unidentified	31.92	**310**	-	+					+			+	+	+				
56	Quercetin-methyl-ether ^b,c^	31.96	-	315.0509	+	tr	tr	-	+	+	+	tr	+	+	+	+	+	+	tr
57	Quercetin-methyl-ether ^b,c^	32.50	-	315.0511	+	-	-	-	tr	+	+	tr	tr	tr	-	+	+	+	tr
58	Unidentified	32.67	-	387.1448	-	-	tr	+	-	-	-	-	-	-	-	-	-	-	-
59	Unidentified	32.93	-	259.1918	-	-	-	+	-	-	-	-	-	-	-	-	-	-	-
60	(*R*/*S*) 1,2-di-*p*-Coumaroylglycerol isomer I ^b,c^	33.03	**312**, 300sh	383.1137	-	-	-	+	-	-	-	-	-	-	-	-	-	-	-
61	Luteolin-5-methyl ether ^b,c^	33.21	350, 298sh, **267**	299.0549	+	+	+	-	+	-	+	+	+	+	+	++	+	++	++
62	Unidentified	33.55	**320**	417.1558	-	-	tr	+	-	-	-	-	-	-	-	-	-	-	-
63	Quercetin-di-methyl-ether ^b,c^	33.91	256, 354	329.0669	+	+	+	+	+	+	+	+	+	+	tr	+	+	+	+
64	1,3-di-*p*-Coumaroylglycerol ^b,c^	33.98	**312**	383.1143	-	tr	+	+	-	tr	-	tr	-	-	-	-	-	-	-
65	(*R*/*S*) 1-*p*-Coumaroyl-3-feruloylglycerol ^b,c^	34.48	**316**	413.1241	-	+	+	+	-	-	-	-	-	-	-	-	-	-	-
66	Galangin-5-methyl-ether ^b,c^	34.58	**353**	283.0612	-	+	+	+	-	-	-	-	-	-	-	-	-	-	-
67	(*R*/*S*) 1,2-di-*p*-Coumaroylglycerol isomer II ^b,c^	34.70	**315**	383.1137	+	+	+	-	+	+	+	+	+	+	tr	+	+	+	+
68	5-Methyl-pinobanksin-3- acetate ^b,c^	34.69	**280**	327.0878	+	+	+	-	+	+	+	+	+	+	tr	+	+	+	tr
69	1,3-di-Feruloylglycerol ^b,c^	34.84	**320**	443.1348	-	-	+	+	-	-	-	-	-	-	-	-	-	-	-
70	2-Acetyl-1,3-di-caffeoylglycerol ^b,c^	35.25	**320**	457.1141	-	+	+	+	-	-	-	tr	-	-	-	-	-	-	-
71	Quercetin-methyl-ether ^b,c^	36.81	**362**	315.0509	+	+	-	-	+	+	+	+	+	+	tr	+	+	+	+
72	Kaempferol-methyl-ether ^b,c^	36.90	-	299.0563	+	+	tr	-	+	+	+	+	+	+	tr	+	+	+	+
73	Caffeic acid butyl or isobutyl ester isomer isomer I ^b,c^	37.46		235.0978	-	-	-	-	+	+	+	+	-	-	-	-		-	-
74	Pinobanksin-3-*O*-hydroxybutyrate or isobutyrate ^b,c^	37.82	**278**	357.0975	+	-	-	-	tr	+	tr	-	tr	tr	-	tr	+	tr	tr
75	Caffeic acid butyl or isobutyl ester isomer II ^b,c^	38.22		235.0976	-	-				+	-	tr	-	-	-	-	-	-	tr
76	* Caffeic acid prenyl ester isomer ^b,c^	38.15	**320**	247.0975	+	+	tr	-	+	tr	+	+	+	+	tr	-	-	-	-
77	* Flavonoid ^b,c^	38.66	-	313.0719	-	-	-	+	-	-	-	-	-	-	-	tr	-	tr	-
78	Quercetin-dimethyl-ether ^b,c^	39.30	**353**	329.0669	+	tr	tr	-	+	+	+	+	+	+		+	+	+	+
79	Caffeic acid 2-methyl-2-butenyl ester ^b,c^	39.50	**325**	247.0979	+	++	+	-	+++	++	++	+++	+++	+++	+++	+++	+	++	++
80	Caffeic acid derivate ^b,c^	40.56	-	269.0817	+	+	tr	-	tr	+	-	tr	+	+	-	tr	tr	tr	tr
81	Caffeic acid 3-methyl-2-butenyl ester (Basic prenyl ester) ^b,c^	40.91	**325**	247.0979	++	++	+	tr	+++	++	++	+++	+++	+++	+++	+++	+++	++	++
82	Caffeic acid 3-methyl-3-butenyl ester ^b,c^	41.42	**325**	247.0977	+	++	+	-	+	+	+	+	+	+	+	tr	+	tr	+
83	(*R*/*S*) 2-Acetyl-1-caffeoyl-3-*p*-coumaroylglycerol ^b,c^	41.91	**315**	441.1197	-	+	+	+	-	-	-	-	-	-	-	-	-	-	-
84	Chrysin ^a,b,c^	42.38	312sh, **268**	253.0505	+++	++	++	-	+++	+++	+++	+++	+++	+++	+++	+++	+++	+++	+++
85	Caffeic acid benzyl ester ^b,c^	42.69	**326**	269.0818	++	++	++	+	++	++	++	++	++	++	++	+	++	++	++
86	(*R*/*S*) 2-Acetyl-1-caffeoyl-3-feruloylglycerol ^b,c^	42.71	**325**	471.1297	-	-	tr	+	-	-	-	-	-	-	-	-	-	-	-
87	* Sakuranetin isomer ^c^	43.29	**287**	285.0769	-	-	-	+	-	-	-	-	-	-	-	-	-	-	-
88	Pinocembrin ^b,c^	43.41	**290**	255.0666	+++	++	++	tr	+++	+++	+++	+++	+++	+++	+++	+++	+++	+++	+++
89	(*R*/*S*) 1-Acetyl-2-caffeoyl-3-feruloylglycerol	43.63	**320**	471.1298	-	tr	tr	+	-	-	-	-	-	-	-	-	-	-	-
90	Sakuranetin ^b,c^	44.69	**290**	285.0773	+	+	+	+	++	++	++	+	+++	+++	+	+	+	+	+
91	Galangin ^a,b,c^	45.17	360, **266**	269.0454	++	+	++	-	+++	+++	+	++	+++	+++	+++	+++	+++	+++	+++
92	Acacetin ^a,b,c^	45.78	335, **269**	283.0614	+	+	++	+	+	+	+	+	+	+	tr	-	+	-	+
93	Ermanin isomer ^b,c^	46.13	333, **275**	313.0721	-	-	++	++	-	-	+	-	-	-	-	-	-	-	-
94	Caffeic acid pentyl or isopentylester ^b,c^	46.82	-	249.1138	-	+	-	-	tr	+	tr	tr	-	-	-	tr	-	tr	-
95	Caffeic acid phenethyl ester (CAPE) ^b,c^	47.21	326	283.0981	++	+	+	-	++	++	++	++	++	++	++	++	+	++	++
96	Pinobanksin 3-*O*-acetate ^b,c^	47.69	**295**	313.0725	+++	++	++	-	+++	+++	+++	+++	+++	+++	+++	+++	+++	+++	+++
97	Kaempferide (Kaempferol 4’-methyl ether) ^b,c^	47.73	**365**, 267	299.0563	-	-	tr	+	tr	-	tr	-	tr	tr	-	-	-	-	tr
98	Methoxychrysin ^b,c^	48.06	310sh, **266**	269.0447	+	+	+	-	+	+	-	+	++	++	-	+	+	+	++
99	Quercetin-dimethyl ether ^b,c^	48.19	**#370**	329.0667	-	-	+	+	-	-	+	-	-	-	-	-		-	-
100	Ermanin (Kaempferol-3,4′-dimethyleter) ^b,c^	30.65	**350**, 267	313.0719	+	-	tr	-	+	+	+	+	+	+	tr	-	+	-	tr
101	*p*-Coumaric acid 3-methyl-3-butenyl ester ^b,c^	50.69	**313**	231.1028	+	+	tr	-	+	+	+	+	+	+	tr	+	+	+	-
102	2-Acetyl-1,3-di-*p*-coumaroylglycerol ^b,c^	50.93	**312**	425.1242	+	+	++	++	-	-	-	-	tr	tr	+	-	-	-	-
103	Ayanin (3,7,4′-Trimethylquercetin) ^b,c^	51.40	271, **334**	343.0822	-	-	tr	+	-	-	-	-	-	-	-	-	-	-	-
104	(*R*/*S*) 2-Acetyl-3-*p*-coumaroyl-1-feruloylglycerol ^b,c^	51.87	**316**	455.1336	+	+	++	++	-	-	-	-	tr	tr	-	-	-	-	-
105	(*R*/*S*) 1-Acetyl-2,3-di-*p*-coumaroylglycerol ^b,c^	51.98	**311**	425.1244	-	tr	tr	+	-	-	-	-	-	-	-	-	-	-	-
106	*p*-Coumaric acid 3-methyl-2-butenyl or 2-methyl-2-butenyl ester ^b,c^	52.18	**313**	231.1027	+	+	+	-	++	++	++	+	++	++	++	+	++	+	++
107	2-Acetyl-1,3-di-feruloylglycerol ^b,c^	52.49	**324**	485.1456	-	+	++	++	-	-	-	-	-	-	-	-	-	-	-
108	(*R*/*S*) 2-Acetyl-3-*p*-coumaroyl-1-feruloylglycerol ^b,c^	52.79	**311**	455.1345	-	+	+	+	-	-	-	-	-	-	-	-	-	-	-
109	(*R*/*S*) 1-Acetyl-2-*p*-coumaroyl-3-feruloylglycerol ^b,c^	53.01	**315**	455.1347	-	-	tr	+	-	-	-	-	-	-	-	-	-	-	-
110	Unidentified	53.54	-	311.2229	+	-	+	-	+	+	+	tr	tr	tr	-	-	-	-	-
111	*p*-Coumaric acid benzyl ester ^b,c^	53.88	**316**	253.0869	+	+	++	++	++	++	++	+	++	++	++	+	+	+	+
112	(*R*/*S*) 1-Acetyl-2,3-di-feruloylglycerol ^b,c^	53.90	**324**	485.1455	-	tr	tr	+	-	-	-	-	-	-	-	-	-	-	-
113	Unidentified	54.17	-	295.0978	-	+	-	-	-	-	-	-	-	-	-	++	-	++	+
114	Unidentified	55.03	**305**	433.0927	+	+	-	-	+	-	-	++	+	+	+	-	-	-	-
115	^iw^ Ferulic acid benzyl ester ^b,c^	55.35	**320**	283.0975	+	+	++	++	+	+	tr	-	tr	tr	+	+	+	+	+
116	Caffeic acid cinnamyl ester ^b,c^	56.10	**323**	295.0982	+	++	++	-	+	+	++	+	++	++	+	+	+	+	++
117	Pinobanksin-3-*O*-propanoate ^b,c^	58.20	**294**	327.0878	+	+	+	-	+	+	+	+	++	++	+	+	+	+	++
118	*p*-Coumaric acid phenethyl ester ^b,c^	58.46	**310**	267.1031	+	+	+	+	+	+	+	+	+	+	tr	+	-	+	-
119	Pinostrobin chalcone ^b,c^	60.56	**343**	269.0827	tr	-	tr	-	+	+	+	tr	tr	tr	tr	-	-	-	-
120	* Flavonoid	62.12	**280**	271.0979	-	-	-	-	+	+	+	-	+	+	-	+	-	+	-
121	^iw^ Tectochrysin	63.00	313, **268**	-	++	+	+	-	++	++	++	++	++	++	++	+	-	+	-
122	^iw^ Pinostrobin	63.48	**288**	-	+	+	+	-	++	++	++	+	++	++	++	+	-	+	-
123	*p*-Coumaric acid cinnamyl ester ^b,c^	64.11	**313**	279.1029	+	++	+	-	+	+	++	+	++	++	+	+	+	+	+
124	Unidentified	64.24	-	321.2439	+	-	-	-	-	-	-	-	-	-	-	-	-	-	-
125	Unidentified	64.41	-	521.2767	-	-	tr	+	-	-	-	-	-	-	-	-	-	-	-
126	Unidentified	64.78	-	551.2874	+	++			++	++	++	-	++	++	++	+	-	+	
127	Unidentified	64.60	**323**	-	-	-	-	+	-	-	-	-	-	-		-	-	-	-
128	Pinobanksin 3-*O*-butanoate or isobutanoate ^b,c^	64.92	**293**	341.1037	+	+	+	-	++	++	++	+	++	++	++	+	++	+	+
129	Pinobanksin 3-*O*-pentenoate or isopentenoate isomer I ^b,c^	65.55	**292**	353.1039	+	tr	+	-	+	+	+	+	+	+	+	+	++	+	+
130	Pinobanksin 3-*O*-pentenoate or isopentenoate isomer II ^b,c^	65.90	**282**	353.1035	+	-	tr	-	+	tr	+	+	+	+	tr	+	tr	+	tr
131	Pinobanksin 3-*O*-benzoate ^b,c^	66.91	**#278**	375.0878	+	-	-	-	tr	tr	+	-	tr	tr	-	tr	-	tr	+
132	Unidentified	67.34	**279**	-			+	++	tr						-	-	-	-	
133	Unidentified	67.77	-	519.3697	-	-	-	+	-	-		-	-	-	-	-	-	-	
134	Pinobanksin 3-*O*-pentanoate or isopentenoate isomer I ^b,c^	67.88	**293**	355.1192	+	+	tr	-	+	++	++	+	++	++	+	+	+	+	tr
135	Pinobanksin 3-*O*-pentanoate or isopentenoateisomer II ^b,c^	68.02	**293**	355.1194	+	-	tr	-	++	-	+	-	-	-	-	tr	+	tr	+
136	Unidentified	68.18	-	315.1606	+	+	-	-	+	+	-	-	+	+	-	-	-	-	-
137	Unidentified	68.23	-	463.3284	+	-	-	-	+	-	-	-	+	+	-	-	-	-	-
138	Pinobanksin 3-*O*-hexenoate or isohexenoate ^c^	68.64	-	367.1189	+	-	tr	-	+	+	tr	-	+	+	-	tr	+	tr	+
139	Unidentified	68.86	-	471.3479	+	-	tr	-	+	-	tr	-	tr	tr	-	-	-	-	tr
140	Pinobanksin-3-*O*-cinnamate ^c^	69.00	**278**	401.1033	+	-	tr	-	tr	-	-	-	-	-	-	-	-	-	tr
141	Pinobanksin-3-*O*-hydroxycinnamate^,c^	69.31	**285**	403.1197	++	++	tr	-	+	+++	++	++	++	++	-	+	+	+	+
142	Metoxycinnamic acid cinnamyl ester ^b,c^	69.35	**282**	293.2125	+	+	++	-	+++	+	+	+	+++	+++	-	++	++	++	+
143	Unidentified	69.66	-	531.3696	-	-	tr	+	-	-		-	-	-	-	-		-	-
144	Pinobanksin 3-*O*-hexanoate or isohexanoate isomer I ^b,c^	69.67	**281**	369.1347	+	-	-	-	+	+	+	-	+	+	-	tr	+	tr	-
145	Unidentified	69.80	-	473.3641	-	-	tr	+	-	-	-	-	-	-	-	-	-	-	-
146	Pinobanksin 3-*O*-hexanoate or isohexanoate isomer II ^b,c^	69.96	**281**	369.1347	+	+	-	-	++	++	++	tr	++	++	++	+	-	+	+
147	Unidentified	70.2	-	533.3855	-	+	-	-	-	-	-	-	-	-		-	-	-	-
148	Unidentified	70.34	-	343.2855	-	-	-	+	-	-	-	-	-	-		-	-	-	-
149	Unidentified	70.73	-	295.2279	+	tr	tr	-	+	+	+	tr	+	+		+	-	+	+
150	Pinobanksin 3-*O*-phenyl pentenoate or phenyl isopentenoate ester ^c^	70.97	**#282**	429.1344	+	-	-	-	+	-	tr	-	-	-	tr	-	-	-	+
151	Unidentified	71.18	-	469.3316	+	+	-	-	+	+	+	tr	+	+	tr	+	+	+	+

Table legend: UV max [nm]—maximum of UV absorption, higher maximum is bolded; ASP—Aspindza; NOR—Norio; PAS—Pasanauri; MES—Mestia; ORG—Orgora; VAR—Vardzia; OTA—Ota; QVA—Qvakhreli U.R.1—unknown region 1; U.R.2—unknown region 2; MTS—Mtskhete; KAK—Kakheti; AKH—Akhatsikhe; DUS—Dusheti; IME—Imereti; #—UV spectrum is weak due to low concentration; ^a^ component identified by comparison with standard; ^b^ component identified by comparison with literature; ^c^ component identified by prediction of mass fragment and UV spectrum; * component tentatively identified; ^iw^ component does not produce or produces low/trace amount of ions in negative mode; - component absent; tr component present in traces; + component present in low amount; ++ component present in average amount; +++ component present in high amount.

**Table 3:** Colorimetric assays (total phenolic and flavonoid content, antiradical and antioxidant activity) and extraction efficiency of Georgian propolis.

Propolis Sample	Extraction Efficiency [%]	TP [mgQE/g]		TF [mgGAE/g]		DPPH [mgGAE/g]		FRAP [mmol Fe^2+^/g]	
		Propolis	Extract	Propolis	Extract	Propolis	Extract	Propolis	Extract
ASP	54.92	93.36 ± 2.74	170.00 ± 5.00	67.16 ± 1.31	122.28 ± 2.39	38.59 ± 0.71	70.26 ± 1.30	5.43 ± 0.00	9.88 ± 0.18
NOR	23.44	27.39 ± 0.91	116.86 ± 3.87	7.62 ± 0.29	32.51 ± 1.25	21.92 ± 0.74	93.53 ± 3.16	2.10 ± 0.00	8.97 ± 0.23
PAS	24.61	28.15 ± 0.85	114.37 ± 3.45	8.57 ± 0.20	34.81 ± 0.79	13.48 ± 0.74	54.79 ± 2.22	2.05 ± 0.00	8.33 ± 0.16
MES	39.45	35.46 ± 1.51	89.88 ± 3.82	7.57 ± 0.19	19.19 ± 0.48	22.19 ± 0.00	56.26 ± 0.82	3.16 ± 0.00	8.01 ± 0.18
ORG	57.93	111.84 ± 1.48	193.06 ± 2.55	55.65 ± 2.74	96.07 ± 4.73	68.19 ± 0.61	117.71 ± 1.04	7.97 ± 0.00	13.76 ± 0.15
VAR	49.07	98.70 ± 1.01	201.15 ± 2.05	61.58 ± 1.41	125.50 ± 2.88	40.33 ± 0.84	82.18 ± 1.72	6.89 ± 0.00	14.03 ± 0.27
OTA	52.23	126.77 ± 1.64	242.71 ± 3.12	63.76 ± 0.82	122.07 ± 1.56	37.22 ± 0.32	71.26 ± 0.60	7.05 ± 0.00	13.51 ± 0.31
QVA	39.46	86.30 ± 1.95	218.70 ± 4.94	32.16 ± 0.57	81.50 ± 1.44	45.92 ± 0.59	116.38 ± 1.50	6.64 ± 0.00	16.83 ± 1.02
U.R.1	39.53	73.13 ± 1.76	185.00 ± 4.43	41.59 ± 1.04	105.22 ± 2.62	43.22 ± 0.24	109.33 ± 0.60	5.01 ± 0.00	12.68 ± 0.51
U.R.2	37.06	66.21 ± 1.40	178.65 ± 3.79	41.46 ± 0.74	111.87 ± 2.00	41.68 ± 0.08	112.46 ± 0.20	4.66 ± 0.00	12.58 ± 0.07
MTS	32.67	60.75 ± 2.33	185.96 ± 7.13	29.49 ± 0.41	90.28 ± 1.25	19.09 ± 0.99	58.44 ± 3.04	3.87 ± 0.00	11.83 ± 0.24
KAK	28.41	32.16 ± 1.24	113.20 ± 4.37	12.73 ± 0.38	44.81 ± 1.33	23.98 ± 1.10	84.41 ± 3.88	2.21 ± 0.00	7.77 ± 0.20
AKH	31.98	56.81 ± 0.74	177.65 ± 2.31	39.61 ± 1.54	123.85 ± 4.81	18.60 ± 1.65	58.16 ± 5.16	3.54 ± 0.00	11.06 ± 0.18
DUS	46.29	78.87 ± 3.23	170.39 ± 6.98	45.99 ± 0.72	99.36 ± 1.56	27.09 ± 1.35	58.53 ± 2.92	6.10 ± 0.00	13.17 ± 0.41
IME	47.55	86.61 ± 2.00	182.14 ± 4.20	53.60 ± 1.63	112.72 ± 3.44	22.77 ± 0.40	47.88 ± 0.83	6.24 ± 0.00	13.13 ± 0.32

Table legend: ASP—Aspindza; NOR—Norio; PAS—Pasanauri; MES—Mestia; ORG—Orgora; VAR—Vardzia; OTA—Ota; QVA—Qvakhreli U.R.1—unknown region 1; U.R.2—unknown region 2; MTS—Mtskhete; KAK—Kakheti; AKH—Akhatsikhe; DUS—Dusheti; IME—Imereti; DPPH—radical scavenging activity in DPPH test; FRAP—ferric reducing antioxidant power; TP—total phenolic content; FC—flavonoid content; [mgGAE/g]—concentration or activity as mg of gallic acid equivalents per gram of crude propolis or its dry extract; FC—flavonoid content; [mgQE/g]—concentration or activity as mg of quercetin per gram of crude propolis or its dry extract.

**Table 4:** Correlation matrices of colorimetric assays.

Correlation Matrix of Crude Propolis				
Variables	TP Propolis	TF Propolis	FRAP Propolis	DPPH Propolis
Extraction efficiency	r = 0.897	r = 0.865	r = 0.894	r = 0.682
	*p* < 0.000	*p* < 0.000	*p* < 0.000	*p* = 0.005
DPPH propolis	r = 0.698	r = 0.568	r = 0.754	-
	*p* = 0.004	*p* = 0.027	*p* < 0.000	-
FRAP propolis	r = 0.955	r = 0.847	-	-
	*p* < 0.000	*p* < 0.000	-	-
TF propolis	r = 0.921	-	-	-
	*p* < 0.000	-	-	-
**Correlation Matrix of Dried Extracts**				
**Variables**	**TP** **Extract**	**TF** **Extract**	**FRAP** **Extract**	**DPPH** **Propolis**
Extraction efficiency	r = 0.600	r = 0.634	r = 0.542	no correlation, *p* > 0.05
	*p* = 0.018	*p* = 0.011	*p* = 0.037	
FRAP extract	r = 0.885	r = 0.653	-	-
	*p* < 0.000	*p* = 0.008	-	-
TF extract	r = 0.834	-	-	-
	*p* < 0.000	-	-	-

Table legends: - lack of correlation.

**Table 5:** Antimicrobial properties of Georgian propolis *.

Sample	GRAM-POSITIVE					GRAM-NEGATIVE			FUNGI			
	*S. aureus* 25923	MLS_b_	MRSA P19	*E. faecalis* 29212	*B. subtilis* 6633	*E. coli* 25922	*K. pneumoniae* 700603	*P. aeruginosa* 27853	*C. albicans* 90028	*C. glabrata* 90030	*C. kruesi* 6258	*S. cerevisiae* 3963
	Disc/MIC *	Disc/MIC *	Disc/MIC *	Disc/MIC *	Disc/MIC *	Disc/MIC *	Disc/MIC *	Disc/MIC *	Disc/MIC *	Disc/MIC *	Disc/MIC *	Disc/MIC *
ASP	17/128	17/128	17/128	10/1024	12/1024	6/>1024	6/>1024	6/>1024	11/1024	11/>1024	11/>1024	12/1024
NOR	14/512	14/512	14/512	9/>1024	10/1024	6/>1024	6/>1024	6/>1024	10/1024	10/>1024	9/>1024	11/>1024
PAS	14/512	13/512	13/512	9/>1024	9/>1024	6/>1024	6/>1024	6/>1024	10/1024	10/>1024	11/1024	11/>1024
MES	14/512	13/512	13/512	9/>1024	9/>1024	6/>1024	6/>1024	6/>1024	11/1024	11/>1024	11/1024	11/1024
ORG	13/512	14/256	18/128	6/>1024	8/>1024	6/>1024	6/>1024	6/>1024	12/512	10/>1024	6/>1024	14/256
VAR	15/128	16/128	16/128	10/1024	12/1024	6/>1024	6/>1024	6/>1024	12/512	12/1024	12/1024	12/1024
OTA	14/512	14/256	18/128	6/>1024	8/>1024	6/>1024	6/>1024	6/>1024	12/512	10/>1024	6/>1024	16/128
QVA	14/256	14/256	18/128	6/>1024	8/>1024	6/>1024	6/>1024	6/>1024	11/512	8/>1024	6/>1024	14/256
U.R.1	15/256	15/256	18/128	6/>1024	8/>1024	6/>1024	6/>1024	6/>1024	12/512	10/1024	6/>1024	20/128
U.R.1	14/256	14/256	14/256	6/>1024	8/>1024	6/>1024	6/>1024	6/>1024	10/512	8/>1024	6/>1024	10/512
MTS	17/64	20/128	19/128	6/>1024	8/>1024	6/>1024	6/>1024	6/>1024	12/512	8/>1024	6/>1024	12/512
KAK	15/128	19/128	20/128	6/>1024	8/1024	6/>1024	6/>1024	6/>1024	13/512	8/>1024	6/>1024	14/256
AKH	15/128	18/128	17/128	6/>1024	8/>1024	6/>1024	6/>1024	6/>1024	14/512	8/>1024	6/>1024	16/256
DUS	16/128	20/128	19/128	6/>1024	8/>1024	6/>1024	6/>1024	6/>1024	12/512	8/>1024	6/>1024	14/256
IME	22/64	19/128	20/128	6/>1024	9/>1024	6/>1024	6/>1024	6/>1024	14/512	8/>1024	6/>1024	15/256
TF extract	r = −0.516 *p* = 0.049	r = −0.758 *p* = 0.001	r = −0.796 *p* < 0.000	NC	NC	NC	NC	NC	r = −0.605 *p* = 0.017	NC	NC	NC
TP extract	NC	r = −0.560 *p* = 0.030	r = −0.748 *p* = 0.001	NC	NC	NC	NC	NC	r = −0.681 *p* = 0.005	r = −0.547 *p* = 0.035	NC	NC

Table legend: Disc/MIC—Kirby–Bauer disc diffusion method/minimal inhibitory concentration; * values of disc diffusion (Kirby–Bauer method) was presented as mm of inhibition diameter zones, while MIC was described in µg/mL. All antibacterial test values was means of three repetitions; NC—no correlation, *p* > 0.05; TF—flavonoid content; TP—total phenolic content; ASP—Aspindza; NOR—Norio; PAS—Pasanauri; MES—Mestia; ORG—Orgora; VAR—Vardzia; OTA—Ota; QVA—Qvakhreli U.R.1—unknown region 1; U.R.2—unknown region 2; MTS—Mtskhete; KAK—Kakheti; AKH—Akhatsikhe; DUS—Dusheti; IME—Imereti.

## Data Availability

Research data is available from authors.

## Sample Availability

Samples of the compounds are not available from the authors.

## References

[1] Kędzia B. (2008). Pochodzenie propolisu w świetle teorii i badań naukowych. Herba Pol..

[2] Simone-Finstrom M., Spivak M. (2010). Propolis and bee health: The natural history and significance of resin use by honey bees. Apidologie.

[3] Bankova V., Bertelli D., Borba R., Conti B.J., da Silva Cunha I.B., Danert C., Eberlin M.N., Falcão S.I., Isla M.I., Moreno M.I.N. (2019). Standard methods for Apis mellifera propolis research. J. Apic. Res..

[4] Kuropatnicki A.K., Szliszka E., Krol W. (2013). Historical aspects of propolis research in modern times. Evid.-Based Complement. Altern. Med..

[5] Isidorov V.A., Szczepaniak L., Bakier S. (2014). Rapid GC / MS determination of botanical precursors of Eurasian propolis. Food Chem..

[6] Isidorov V.A., Bakier S., Pirożnikow E., Zambrzycka M., Swiecicka I. (2016). Selective behaviour of honeybees in acquiring European propolis plant precursors. J. Chem. Ecol..

[7] Bankova V. (2005). Chemical diversity of propolis and the problem of standardization. J. Ethnopharmacol..

[8] Okinczyc P., Szumny A., Szperlik J., Kulma A., Franiczek R., Zbikowska B., Krzyzanowska B., Sroka Z. (2018). Profile of polyphenolic and essential oil composition of polish propolis, black poplar and aspens buds. Molecules.

[9] Kujumgiev A., Tsvetkova I., Serkedjieva Y., Bankova V., Christov R., Popov S. (1999). Antibacterial, antifungal and antiviral activity of propolis of different geographic origin. J. Ethnopharmacol..

[10] Widelski J., Okińczyc P., Paluch E., Mroczek T., Szperlik J., Żuk M., Sroka Z., Sakipova Z., Chinou I., Skalicka-Woźniak K. (2022). The antimicrobial properties of poplar and aspen–poplar propolises and their active components against selected microorganisms, including Helicobacter pylori. Pathogens.

[11] Białobok S., Bugała W., Hejnowicz A., Jakuszewski T., Jankiewicz L.J., Obmiński Z., Siwecki R., Środoń A., Surmiński J., Suszka B., Białobok S. (1973). Nasze Drzewa Leśne: Topole Populus L..

[12] Šiler B., Skorić M., Mišić D., Kovačević B., Jelić M., Patenković A., Novičić Z.K. (2014). Variability of European Black Poplar (Populus nigra L.) in the Danube Basin.

[13] Isidorov V.A., Wyd I. (2007). Alchemy of Bees. Bees and Bee Products in Chemist Eyes (Alchemia pszczół. Pszczoły i Produkty Pszczele Oczami Chemika).

[14] *Populus nigra*. European Black Poplar. EUFORGEN—The European Forest Genetic Resources Programme. https://www.euforgen.org/species/populus-nigra/.

[15] *Populus tremula*. Eurasian Aspen. EUFORGEN—The European Forest Genetic Resources Programme. https://www.euforgen.org/species/populus-tremula/.

[16] *Betula pubescens*. Downy Birch. EUFORGEN—The European Forest Genetic Resources Programme. https://www.euforgen.org/species/betula-pubescens/.

[17] *Betula pendula*. Silver Birch. EUFORGEN—The European Forest Genetic Resources Programme. https://www.euforgen.org/species/betula-pendula/.

[18] Petropoulou A., Chinou I., Graikou K. (2018). Qualitative analysis and biological evaluation of propolis from Armenia and Georgia. J. Apither. Nat..

[19] Gabunia K. (2016). Optical density and antimicrobial characteristics of Georgian propolis. J. Pharm. Pharmacol..

[20] Aladashvili N., Kunchulia-Gurashvili L., Imnadze L., Lekishvili N., Nizharadze N. (2019). Study of correlation of polyphenolic content and antioxidant activity of Georgian propolis. TSMU Collect. Sci. Work..

[21] Ciochoń M. (2022). Phytochemical Analysis of Georgian and Kazakh Propolis. Master’s Thesis.

[22] Okińczyc P., Widelski J., Szperlik J., Żuk M., Mroczek T., Skalicka-Woźniak K., Sakipova Z., Widelska G., Kuś P.M. (2021). Impact of plant origin on eurasian propolis on phenolic profile and classical antioxidant activity. Biomolecules.

[23] Svečnjak L., Marijanović Z., Okińczyc P., Kuś P.M., Jerković I. (2020). Mediterranean propolis from the adriatic sea islands as a source of natural antioxidants: Comprehensive chemical biodiversity determined by GC-MS, ftiratr, UHPLC-DAD-QQTOF-MS, DPPH and FRAP assay. Antioxidants.

[24] Woo S.O., Hong I., Han S. (2015). Extraction Properties of Propolis with Ethanol Concentration. J. Apic..

[25] Grecka K., Kuś P.M., Okińczyc P., Worobo R.W., Walkusz J., Szweda P. (2019). The anti-staphylococcal potential of ethanolic Polish propolis extracts. Molecules.

[26] Smith C.A., O’Maille G., Want E.J., Qin C., Trauger S.A., Brandon T.R., Custodio D.E., Abagyan R., Siuzdak G. (2005). METLIN: A metabolite mass spectral database. Ther. Drug Monit..

[27] Popova M., Giannopoulou E., Skalicka-Wózniak K., Graikou K., Widelski J., Bankova V., Kalofonos H., Sivolapenko G., Gaweł-Bȩben K., Antosiewicz B. (2017). Characterization and biological evaluation of propolis from Poland. Molecules.

[28] Christov R., Trusheva B., Popova M., Bankova V., Bertrand M. (2006). Chemical composition of propolis from Canada, its antiradical activity and plant origin. Nat. Prod. Res..

[29] Paluch E., Okińczyc P., Zwyrzykowska-Wodzińska A., Szperlik J., Żarowska B., Duda-Madej A., Bąbelewski P., Włodarczyk M., Wojtasik W., Kupczyński R. (2021). Composition and antimicrobial activity of Ilex leaves water extracts. Molecules.

[30] Isidorov V.A., Brzozowska M., Czyzewska U., Glinka L. (2008). Gas chromatographic investigation of phenylpropenoid glycerides from aspen (*Populus tremula* L.) buds. J. Chromatogr. A.

[31] Saleh K., Zhang T., Fearnley J., George Watson D. (2015). A comparison of the constituents of propolis from different regions of the United Kingdom by liquid chromatography-high resolution mass spectrometry using a metabolomics approach. Curr. Metab..

[32] Tian Y., Liimatainen J., Alanne A.L., Lindstedt A., Liu P., Sinkkonen J., Kallio H., Yang B. (2017). Phenolic compounds extracted by acidic aqueous ethanol from berries and leaves of different berry plants. Food Chem..

[33] Mai F., Glomb M.A. (2013). Isolation of phenolic compounds from iceberg lettuce and impact on enzymatic browning. J. Agric. Food Chem..

[34] Sonkamble V.V., Kamble L.H. (2015). Antidiabetic potential and identification of phytochemicals from Tinospora cordifolia phytochemicals as glucosidase and amylase inhibitors view project protease inhibitors view project. Am. J. Phytomed. Clin. Ther..

[35] Popova M., Trusheva B., Khismatullin R., Gavrilova N., Legotkina G., Lyapunov J., Bankova V. (2013). The triple botanical origin of Russian propolis from the Perm Region, its phenolic content and antimicrobial activity. Nat. Prod. Commun..

[36] Rubiolo P., Casetta C., Cagliero C., Brevard H., Sgorbini B., Bicchi C. (2013). *Populus nigra* L. bud absolute: A case study for a strategy of analysis of natural complex substances. Anal. Bioanal. Chem..

[37] Coleman G.D., Ernst S.G. (1990). Phenolic composition of bud exudates of Populus deltoides. Plant Sci..

[38] Pellati F., Orlandini G., Pinetti D., Benvenuti S. (2011). HPLC-DAD and HPLC-ESI-MS/MS methods for metabolite profiling of propolis extracts. J. Pharm. Biomed. Anal..

[39] Arafa M.G., Ghalwash D., El-Kersh D.M., Elmazar M.M. (2018). Propolis-based niosomes as oromuco-adhesive films: A randomized clinical trial of a therapeutic drug delivery platform for the treatment of oral recurrent aphthous ulcers. Sci. Rep..

[40] Gardana C., Scaglianti M., Pietta P., Simonetti P. (2007). Analysis of the polyphenolic fraction of propolis from different sources by liquid chromatography-tandem mass spectrometry. J. Pharm. Biomed. Anal..

[41] Shi H., Yang H., Zhang X., Yu L. (2012). Identification and quantification of phytochemical composition and anti-inflammatory and radical scavenging properties of methanolic extracts of Chinese propolis. J. Agric. Food Chem..

[42] Mori A., Nishino C., Enoki N., Tawata S. (1987). Antibacterial activity and mode of action of plant flavonoids against Proteus vulgaris and Staphylococcus aureus. Phytochemistry.

[43] Prenyl Caffeate. Compound Summmary. The National Center for Biotechnology Information (NCBI) Website. https://pubchem.ncbi.nlm.nih.gov/compound/Prenyl-caffeate.

[44] Caffeic Acid Isoprenyl Ester. Compound Summary. The National Center for Biotechnology Information (NCBI) Website. https://pubchem.ncbi.nlm.nih.gov/compound/Caffeic-acid-isoprenyl-ester.

[45] Mavri A., Abramovič H., Polak T., Bertoncelj J., Jamnik P., Smole Moažina S., Jeršek B. (2012). Chemical properties and antioxidant and antimicrobial activities of slovenian propolis. Chem. Biodivers..

[46] Gardana C., Simonetti P. (2011). Evaluation of allergens in propolis by ultra-performance liquid chromatography/tandem mass spectrometry. Rapid Commun. Mass Spectrom..

[47] Greenaway W., May J., Scaysbrook T., Whatley F.R. (1991). Identification by gas chromatography-mass spectrometry of 150 compounds in propolis. Z. Naturforsch.-Sect. C J. Biosci..

[48] Asakawa Y., Takemoto T., Wollenweber E., Aratani T. (1977). Lasiocarpin A, B and C, three novel phenolic triglycerides from Populus lasiocarpa. Phytochemistry.

[49] Boisard S., Le Ray A.M., Gatto J., Aumond M.C., Blanchard P., Derbré S., Flurin C., Richomme P. (2014). Chemical composition, antioxidant and anti-AGEs activities of a French poplar type propolis. J. Agric. Food Chem..

[50] Wang K., Zhang J., Ping S., Ma Q., Chen X., Xuan H. (2014). Anti-inflammatory effects of ethanol extracts of Chinese propolis and buds from poplar ( *Populus × canadensis* ). J. Ethnopharmacol..

[51] Amorati R., Valgimigli L. (2015). Advantages and limitations of common testing methods for antioxidants. Free Radic. Res..

[52] da Silva C., Prasniewski A., Calegari M.A., de Lima V.A., Oldoni T.L.C. (2018). Determination of total phenolic compounds and antioxidant activity of ethanolic extracts of propolis using ATR–FT-IR spectroscopy and chemometrics. Food Anal. Methods.

[53] Marghitas L.A., Dezmirean D., Moise A., Mihai C.M., Laslo L.S. (2009). DPPH method for evaluation of propolis antioxidant activity. Bull. UASVM Anim. Sci. Biotechnol..

[54] Lima B., Tapia A., Luna L., Fabani M.P., Schmeda-Hlrschmann G., Podio N.S., Wunderlin D.A., Feresin G.E. (2009). Main flavonoids, DPPH activity, and metal Content allow determination of the geographical origin of propolis from the province of San Juan (Argentina). J. Agric. Food Chem..

[55] Seyoum A., Asres K., El-Fiky F.K. (2006). Structure-radical scavenging activity relationships of flavonoids. Phytochemistry.

[56] Kaleta J. (2007). The Physicochemical Analysis of Propolis and the Possibility of Its Standardization; in Polish: Analiza Fizykochemiczna Propolisu I Możliwości Jego Standaryzacji. Ph.D. Thesis.

[57] Popova M.P., Bankova V.S., Bogdanov S., Tsvetkova I., Naydenski C., Marcazzan G.L., Sabatini A.G. (2007). Chemical characteristics of poplar type propolis of different geographic origin. Apidologie.

[58] Bonvehí J.S., Gutiérrez A.L. (2012). The antimicrobial effects of propolis collected in different regions in the Basque Country (Northern Spain). World J. Microbiol. Biotechnol..

[59] Chaillou L.L., Nazareno M.A. (2009). Bioactivity of propolis from Santiago del Estero, Argentina, related to their chemical composition. Lwt.

[60] Martinotti S., Ranzato E. (2015). Propolis: A new frontier for wound healing?. Burn. Trauma.

[61] (2012). M07-A9 Methods for Dilution Antimicrobial Susceptibility Tests for Bacteria That Grow Aerobically. Approved Standard.

